# Sugar signaling modulates SHOOT MERISTEMLESS expression and meristem function in *Arabidopsis*

**DOI:** 10.1073/pnas.2408699121

**Published:** 2024-09-06

**Authors:** Filipa L. Lopes, Pau Formosa-Jordan, Alice Malivert, Leonor Margalha, Ana Confraria, Regina Feil, John E. Lunn, Henrik Jönsson, Benoît Landrein, Elena Baena-González

**Affiliations:** ^a^Instituto Gulbenkian de Ciência, Oeiras 2780-156, Portugal; ^b^Instituto de Tecnologia Química e Biológica, Universidade Nova de Lisboa, Oeiras 2780-157, Portugal; ^c^Sainsbury Laboratory, University of Cambridge, Cambridge CB2 1LR, United Kingdom; ^d^Department of Biology, University of Oxford, Oxford OX1 3RB, United Kingdom; ^e^Max Planck Institute for Plant Breeding Research, Cologne D-50829, Germany; ^f^Laboratoire Reproduction et Développement des Plantes, Université de Lyon, École Normale Supérieure de Lyon, Université Claude Bernard Lyon 1, CNRS, Institut National de la Recherche Agronomique, Lyon Cedex 07 69342, France; ^g^Max Planck Institute of Molecular Plant Physiology, Potsdam-Golm 14476, Germany; ^h^Department of Applied Mathematics and Theoretical Physics, University of Cambridge, Cambridge CB3 0DZ, United Kingdom; ^i^Computational Biology and Biological Physics, Lund University, Lund 223 62, Sweden

**Keywords:** shoot apical meristem, plant development, sugar signaling, *Arabidopsis thaliana*

## Abstract

In plants, development of all above-ground tissues relies on the shoot apical meristem (SAM) which balances cell proliferation and differentiation to allow life-long growth. To maximize fitness and survival, meristem activity is adjusted to the prevailing conditions through a poorly understood integration of developmental signals with environmental and nutritional information. Here, we show that sugar signals influence SAM function by altering the protein levels of SHOOT MERISTEMLESS (STM), a key regulator of meristem maintenance. STM is less abundant in inflorescence meristems with lower sugar content, resulting from plants being grown or treated under limiting light conditions. Additionally, sucrose but not light is sufficient to sustain STM accumulation in excised inflorescences. Plants overexpressing the α1-subunit of SUCROSE-NON-FERMENTING1-RELATED KINASE 1 (SnRK1) accumulate less STM protein under optimal light conditions, despite higher sugar accumulation in the meristem. Furthermore, SnRK1α1 interacts physically with STM and inhibits its activity in reporter assays, suggesting that SnRK1 represses STM protein function. Contrasting the absence of growth defects in SnRK1α1 overexpressors, silencing *SnRK1α* in the SAM leads to meristem dysfunction and severe developmental phenotypes. This is accompanied by reduced *STM* transcript levels, suggesting indirect effects on STM. Altogether, we demonstrate that sugars promote STM accumulation and that the SnRK1 sugar sensor plays a dual role in the SAM, limiting STM function under unfavorable conditions but being required for overall meristem organization and integrity under favorable conditions. This highlights the importance of sugars and SnRK1 signaling for the proper coordination of meristem activities.

## Significance

The shoot apical meristem (SAM) generates all the aboveground plant organs and is hence crucial for plant adaptation to the environment. However, little is known of how the SAM perceives environmental information and how this impacts meristem activity and plant growth. Here, we show that sugars promote the accumulation of SHOOT MERISTEMLESS (STM), a transcription factor necessary for stem cell identity and proliferation. This is counteracted by SUCROSE NON-FERMENTING1-RELATED KINASE 1 (SnRK1), which is activated when sugar levels decline, and interacts with STM. On the other hand, silencing SnRK1 in the SAM showed that it is needed for meristem integrity. Overall, our data support a dual function for SnRK1 in plant growth and a need to finely balance its activity.

The developmental plasticity of plants is the cornerstone of their evolutionary success. This plasticity relies on continuous organ production by pluripotent stem cells in the meristems. The shoot apical meristem (SAM) generates all above-ground organs and is organized into tightly interconnected functional domains to ensure meristem integrity and maintenance (1). Imbalances between stem cell proliferation and differentiation impair the formation of new organs when stem cells become depleted or cause gross developmental defects (known as fasciation) when stem cells undergo uncontrolled growth (1, 2).

The renewal of stem cells and their pluripotency is ensured by a negative feedback loop between two mobile factors, the WUSCHEL (WUS) transcription factor (TF) and the CLAVATA3 (CLV3) peptide [reviewed in ref. 3]. Meristematic activity is also regulated by SHOOT MERISTEMLESS (STM), another TF essential for the establishment of the SAM and its maintenance (4, 5). Loss-of-function *stm* mutants show growth arrest at the seedling stage due to depletion of the stem cells (4–6). In addition, the most severely affected mutants like *stm-1* display fusions of cotyledons and other organs, indicating a role for STM also in boundary specification (7). STM suppresses differentiation and promotes cell division by inducing the expression of *CYCLIN D3* (*CYCD3*) and *ISOPENTENYL TRANSFERASE7* (*IPT7*), which encodes a key enzyme involved in cytokinin (CK) biosynthesis (8–11). CKs, in turn, are involved in stem cell maintenance, influencing SAM size and organ formation through WUS and STM (12–14). Recent work revealed that STM heterodimerizes with WUS, enhancing WUS binding to the *CLV3* promoter and *CLV3* expression, and repressing stem cell differentiation (15). Conversely, WUS is required for the expression of *STM,* which thereby enhances WUS-mediated stem cell activity (15). STM is also regulated through an interaction with BELL1-LIKE HOMEODOMAIN (BLH) proteins (16) and the formation of these heterodimeric complexes is essential for STM nuclear localization (17, 18). Furthermore, the BLH proteins PENNYWISE (PNY), POUNDFOOLISH (PNF), and *ARABIDOPSIS THALIANA* HOMEOBOX GENE1 (ATH1) contribute redundantly with STM to meristem initiation and maintenance (19).

Because of their sessile lifestyle, plants continuously adjust their development to changes in the environment, and this is reflected in the dynamic nature of the SAM. In addition to its maintenance by a network of TFs and hormonal signals, the SAM also responds to environmental cues that influence the relative size of its subdomains and the type and number of organs it produces. One of the external factors that affect meristem activity is light, which can exert a direct effect through photoreceptor-mediated signaling and an indirect effect by driving photosynthesis and sugar production (20–22). Both light and metabolic signals activate the TARGET OF RAPAMYCIN (TOR) protein kinase, which in turn promotes cell proliferation in the SAM via an increase in the expression of S-phase genes (23, 24). In addition, TOR induces *WUS* expression, partially through an effect on CK degradation (22, 25).

TOR activity is often antagonized by SUCROSE NON-FERMENTING1-RELATED KINASE 1 (SnRK1) which, like TOR, translates environmental information into metabolic and developmental adaptations (26–28). SnRK1 is a heterotrimeric protein kinase complex, composed of an α-catalytic subunit and two regulatory β- and γ-subunits. In *Arabidopsis*, the α-subunit is present in two major isoforms, SnRK1α1 and SnRK1α2 (also known as KIN10 and KIN11) (28). The SnRK1 complex is activated under low carbon conditions to promote energy-saving and nutrient remobilization strategies, while TOR is activated in response to nutrient abundance to promote cell proliferation and growth (26–28).

Despite the importance of STM for establishing and maintaining SAM function, little is known of its potential regulation by environmental signals. Here, we make use of plants expressing transcriptional and translational STM reporters to investigate this question. We show that STM protein accumulation does not respond to CK but that it is clearly induced by photosynthesis-derived sugars. We also show that suboptimal light conditions activate the SnRK1 kinase in the SAM and that SnRK1 interacts with STM and represses its function. Finally, we show that, despite being generally considered a growth repressor, SnRK1 is necessary under favorable conditions to maintain meristem organization and integrity.

## Results

### Light Promotes STM Protein Accumulation.

**Fig. 1.**
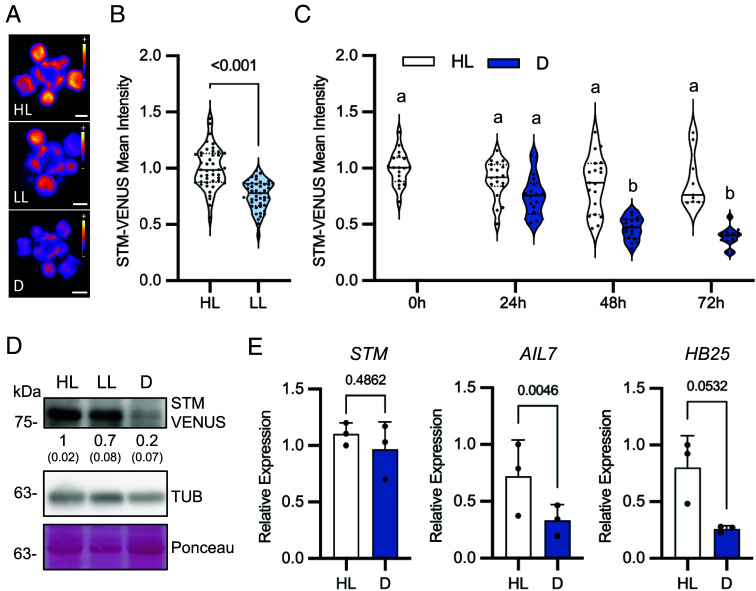
Effect of light on STM expression. (*A–C*), STM-VENUS expression in SAMs of *pSTM::STM-VENUS* plants grown under HL (170 μmol m^−2^ s^−1^) or LL (60 μmol m^−2^ s^−1^) conditions or transferred from HL to darkness (D) or kept under HL for the indicated times. (*A*) Representative STM-VENUS images of SAMs from HL and LL-grown plants and of plants transferred to D for 48 h. (Scale bar, 50 µm.) (*B* and *C*) Quantification of STM-VENUS signal. (*B*) Plots show SAM measurements of plants grown as three independent batches normalized by the mean of the HL condition of each batch (HL, *n* = 44; LL, *n* = 45). Student’s *t* test (*P*-value shown). (*C*) Plots show SAM measurements of plants grown as two to three independent batches normalized by the mean of the HL condition of each batch (0 h, *n* = 18; 24 h L, *n* = 19; 24 h D, *n* = 18; 48 h L, *n* = 19; 48 h D, *n* = 18; 72 h L, *n* = 9; 72 h D, *n* = 12). The 0 h sample serves as control for both L and D treatments. Different letters indicate statistically significant differences (Kruskal–Wallis with Dunn's test; *P* < 0.05). (*D*) Immunoblot analyses of STM and TUBULIN (TUB) protein levels in SAMs of *pSTM::STM-VENUS* plants grown under HL or LL conditions or grown in HL and transferred to D for 48 h. Ponceau staining serves as loading control. Numbers refer to mean STM-VENUS amounts in LL and D as compared to HL (*n* = 2; each a pool of five SAMs; in parentheses, SEM). (*E*) RT-qPCR analyses of *STM* and STM target genes *AIL7* and *HB25* in SAMs of *pSTM::STM-VENUS* plants grown in HL and transferred to D or kept in HL for 48 h. Graphs show the average of three independent samples, each consisting of a pool of five SAMs. Paired ratio *t* test (*P*-values shown).

Light is essential for proper plant development and physiology. To investigate a potential regulatory role of light on STM levels, we made use of an *Arabidopsis* (Col-0) reporter line in which a fluorescently tagged form of the STM protein (STM-VENUS) is expressed under the control of the *STM* promoter [*pSTM::STM-VENUS* (29, 30)]. We measured STM-VENUS levels in inflorescence meristems from 5-wk-old plants grown under, or transiently treated with different light conditions. In one set of experiments, we compared STM-VENUS levels between plants grown under two different irradiances [60 vs. 170 μmol m^−2^ s^−1^, referred to as low light (LL) and high light (HL), respectively]. Irradiance had a strong impact on STM accumulation, with the mean STM-VENUS levels of plants grown under LL being 76% of those grown under HL (Fig. 1 *A* and *B*). In a second set of experiments, we compared STM-VENUS levels between HL-grown plants transferred to darkness for up to 72 h and their corresponding controls maintained under HL conditions. Incubation under darkness had a very severe impact on STM accumulation, with STM-VENUS levels decreasing to 39% over the course of the 72 h dark treatment (Fig. 1 *A* and *C*).

To assess whether the impact of light on STM levels was a general effect on protein abundance in meristems, we extracted total proteins from SAMs of plants constantly grown under HL, LL, or treated with 48 h of darkness and compared STM levels to those of the reference protein TUBULIN (TUB) by immunoblotting (Fig. 1*D*). These analyses confirmed the microscopy results regarding STM-VENUS accumulation, showing that, in LL and dark-treated plants, STM levels were 71% and 23%, respectively, of the STM levels in HL. The immunoblots revealed no impact of the light conditions on TUB accumulation, indicating that the lower STM levels were not caused by a general decrease in protein accumulation. Finally, to assess whether low STM accumulation could be due to reduced *STM* transcript abundance, we dissected SAMs of plants kept under HL conditions or subjected to 48 h darkness and analyzed *STM* transcript levels by qPCR. *STM* levels were not significantly affected by the dark treatment (Fig. 1*E*), showing that the differences in protein accumulation are not due to changes in *STM* transcription or transcript stability. On the other hand, the levels of *AINTEGUMENTA-LIKE 7* (*AIL7*) and *HOMEOBOX PROTEIN 25* (*HB25*), two known gene targets of STM (10), were reduced upon dark treatment (Fig. 1*E*). This is also consistent with the lower STM-VENUS abundance and indicates decreased STM activity in the SAM in these conditions.

### The Response of STM to Light is CK-Independent and Involves Sugars.

Several lines of evidence suggest that *STM* expression could be, like *WUS*, directly regulated by CK (12, 31). To investigate whether CK could also regulate STM at the protein level and hence be involved in the response of STM to light, we first tested whether light influenced CK signaling in inflorescence meristems. To this end, we used plants expressing the synthetic CK reporter *pTCSn::GFP* (32) in similar experiments as described for the *STM-VENUS* reporter line. In plants grown in LL or subjected to a 48 h dark treatment, *pTCSn::GFP* levels were 74% (*SI Appendix*, Fig. S1 *A* and *B*) and 46% (*SI Appendix*, Fig. S1 *A* and *C*) of those in HL plants, respectively. These observations show that CK signaling in inflorescence meristems is, like in vegetative meristems (22), affected by light. We next examined whether CK could impact STM levels in inflorescence meristems. For this, we excised SAMs of HL-grown *STM-VENUS* plants and maintained them under HL in vitro (14) for different periods of time in the absence or presence of 500 nM 6-benzylaminopurine (BAP), a synthetic CK. Dissection of the SAMs led to a strong reduction of the STM-VENUS (*SI Appendix*, Fig. S1*D*) and the *pTCSn::GFP* (*SI Appendix*, Fig. S1*E*) reporter signals, as previously described for the *pTCSn::GFP* and *pWUS::GFP* reporters (14). However, in contrast to *pTCSn::GFP* [*SI Appendix*, Fig. S1*E*; (14)], CK could not sustain STM-VENUS levels (*SI Appendix*, Fig. S1*D*), indicating that the effect of light on STM-VENUS is likely CK-independent.

**Fig. 2.**
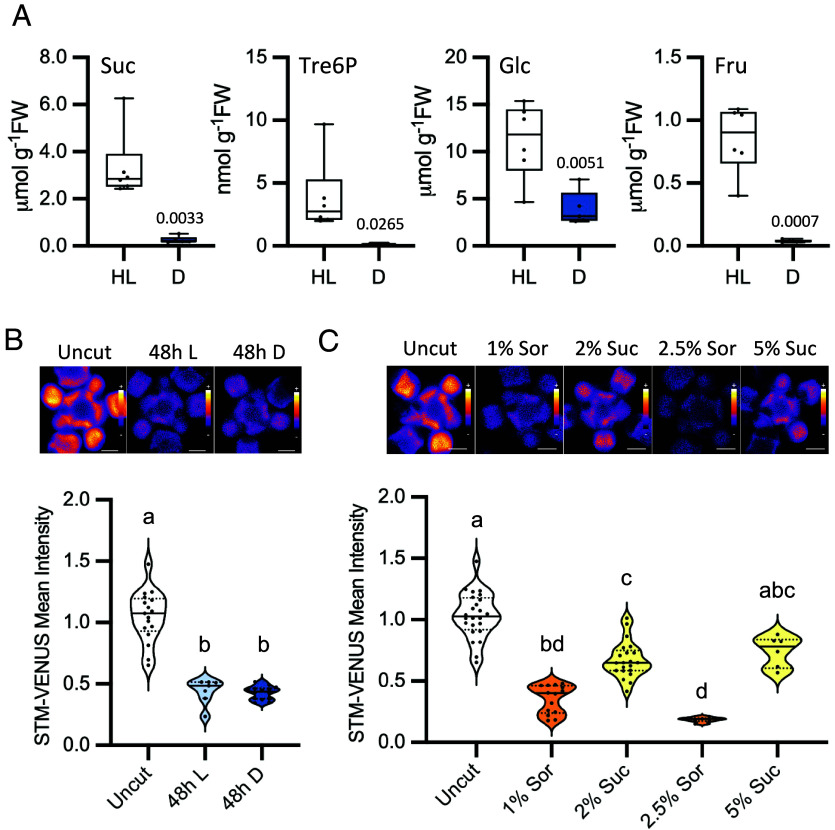
Effect of sugars on STM levels. (*A*) Effect of light on the levels of soluble sugars in SAMs of *pSTM::STM-VENUS* plants grown in HL and transferred to darkness (D) or kept in HL for 48 h. Suc, sucrose; Tre6P, trehalose 6-phosphate; Glc, glucose; Fru, fructose. Plots show measurements of five to six samples, each consisting of a pool of five SAMs from plants grown as two independent batches. Welch’s *t* test (*P*-value shown). (*B*) Effect of light on STM-VENUS levels in cut inflorescences. Inflorescences of *pSTM::STM-VENUS/pSTM::TFP-N7* plants grown under HL were cut and placed in medium without sugar for 48 h under HL (L) or dark (D) conditions, after which the SAMs were dissected and imaged (VENUS). *Upper* panel, representative STM-VENUS images of SAMs. (Scale bar, 50 µm.) *Lower* panel, plots showing SAM measurements of plants grown as one to two independent batches normalized by the mean of the uncut condition of each batch (uncut, *n* = 31, two batches; 48 h L, *n* = 14, one batch; 48 h D, *n* = 21, two batches). Different letters indicate statistically significant differences (Kruskal–Wallis with Dunn's test; *P* < 0.05). (*C*) Effect of sugar on STM-VENUS levels in cut inflorescences. Inflorescences of *pSTM::STM-VENUS/pSTM::TFP-N7* plants grown under HL condition were cut and placed under darkness for 48 h in medium with sucrose (Suc; 2% and 5%) or sorbitol (Sor; 1% and 2.5%) as osmotic control. SAMs were thereafter dissected and imaged (VENUS). *Upper* panel, representative STM-VENUS images of SAMs. (Scale bar, 50 µm.) *Lower* panel, plots showing SAM measurements of plants grown as one to three independent batches normalized by the mean of the uncut condition of each batch (uncut, *n* = 31, three batches; 1% Sor, *n* = 21, two batches; 2% Suc, *n* = 28, three batches; 2.5% Sor, *n* = 7, one batch; 5% Suc, *n* = 6, one batch). Different letters indicate statistically significant differences (Kruskal–Wallis with Dunn's test; *P* < 0.05).

Light plays direct signaling functions through various photoreceptors but also signals indirectly through sugars produced by photosynthesis. We therefore wondered whether the effect of light on STM was direct or mediated by sugars. To investigate this, we first measured the levels of sucrose, glucose, and fructose in the rosettes (*SI Appendix*, Fig. S2*A*) and SAMs (Fig. 2*A*) of HL- and dark-treated plants. We also measured the levels of Tre6P, a regulatory sugar-phosphate that reflects the sucrose status, and that is crucial for sucrose homeostasis, growth promotion, and developmental progression (33). In the light, the levels of sucrose, glucose, and Tre6P were, respectively, 2.1-, 2.2-, and 7.9-fold higher in the SAM than in the rosette. Fructose accumulated to comparable levels in the two organs (Fig. 2*A* and *SI Appendix*, Fig. S2*A*). Incubation in the dark led to a marked depletion of sucrose and fructose both in rosettes (15% and 11% of the levels in the light, respectively) and SAMs (8% and 4% of the levels in the light, respectively), with a much milder reduction being observed for glucose, the most abundant sugar in the SAM (44% and 35% of the levels in the light in rosettes and SAMs, respectively). Tre6P levels were also much lower in dark-treated plants (11% and 3% of the levels in the light in rosettes and SAMs, respectively), reflecting the drop in sucrose levels. To further distinguish between a light and a sugar effect, we excised inflorescences at around 3 cm from the apex and placed them for 48 h in liquid medium. Similarly to what was observed in dissected SAMs (*SI Appendix*, Fig. S1*D*), STM-VENUS signal decreased markedly in cut inflorescences as compared to the uncut controls (Fig. 2*B*). Furthermore, light alone was not sufficient to sustain STM-VENUS expression, as the signal was comparable in cut inflorescences incubated in the light and in the dark (47% and 43% of the levels in the uncut control, respectively). These results, obtained with a double reporter line (*pSTM::STM-VENUS/pSTM::TFP-N7*; Fig. 2*B*), were similar to those obtained for plants expressing STM-VENUS alone (*SI Appendix*, Fig. S2*B*).

To test whether the decrease in STM levels was due to sugar deprivation, we first incubated (48 h in darkness) excised inflorescences of the double marker line in medium supplemented with increasing concentrations of sucrose. Sorbitol, which is not a readily metabolized carbon source, was used as an osmotic control. Sucrose was able to sustain STM-VENUS accumulation, and its effect was largely dose-dependent, leading to STM-VENUS levels close to those of uncut inflorescences when supplied at a 5% concentration (72% of the uncut control values as compared to 18% in the corresponding 2.5% sorbitol control; Fig. 2*C*). STM-VENUS levels did not increase in response to sorbitol, indicating that the effects of sucrose were not osmotic. Similar results were obtained for the single STM-VENUS reporter line (*SI Appendix*, Fig. S2*C*). To test whether the observed effects were transcriptional, we monitored the activity of the *pSTM::TFP-N7* transcriptional reporter. Quantification of the *pSTM::TFP-N7* signal revealed no significant repression of *STM* promoter activity upon inflorescence excision and incubation in darkness (*SI Appendix*, Fig. S2*D*), consistent with the results obtained by qRT-PCR in intact plants (Fig. 1*E*). In addition, incubation in sucrose or sorbitol-containing media had minor effects on TFP levels (*SI Appendix*, Fig. S2*E*) as compared to STM-VENUS (Fig. 2*C*), with the most severe condition (2.5% sorbitol) leading to 65% of the signal in the uncut control as compared to the 18% of the equivalent STM-VENUS samples. This indicates that the effect of sugar deprivation on STM levels does not rely on transcriptional regulation of *STM*.

### The SnRK1 Sugar Sensor Is Expressed in the SAM and Influences STM Levels.

**Fig. 3.**
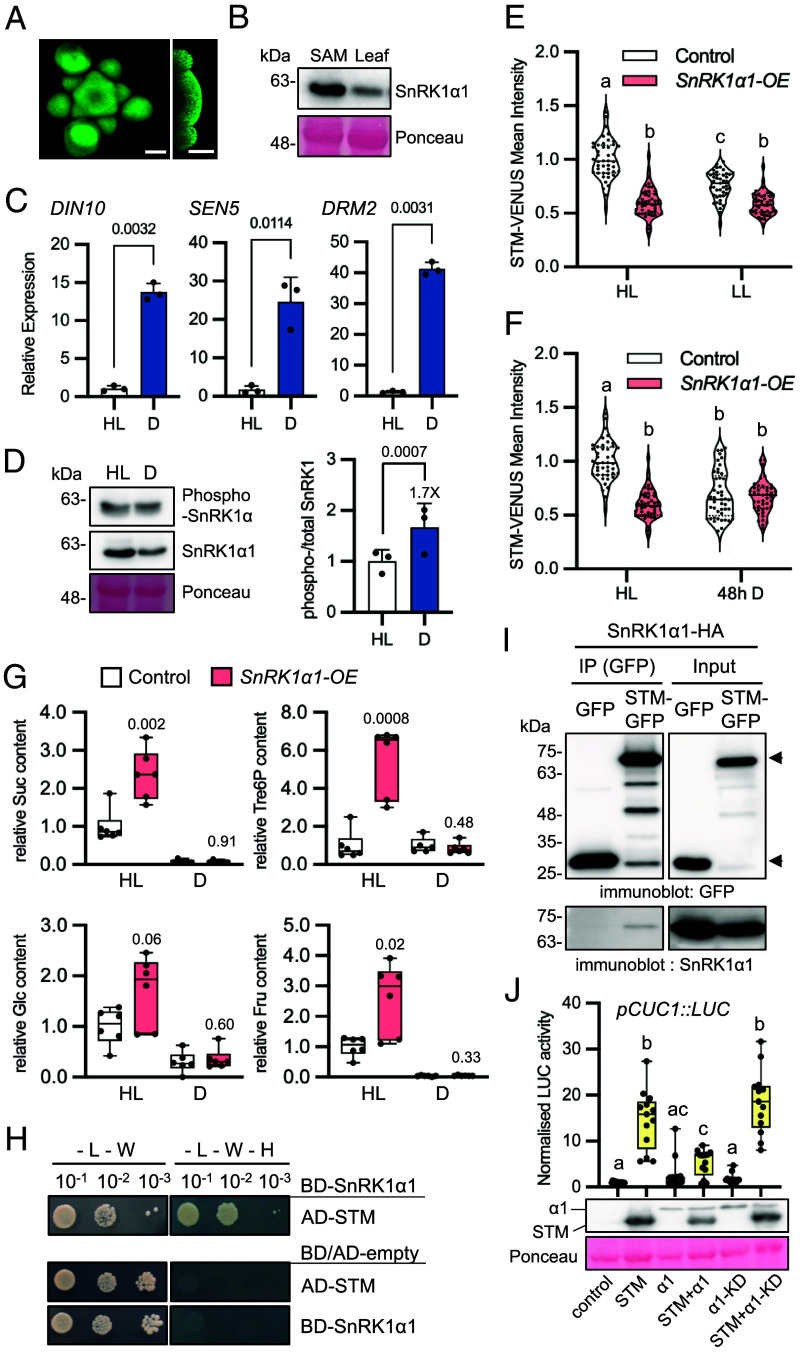
SnRK1 is expressed in the SAM and affects STM response to light. (*A*) SnRK1α1-GFP imaging in the SAM. *Right* panel, SAM longitudinal section. (Scale bars, 50 µm.) (*B*) Immunoblot analyses of SnRK1α1 in SAMs and rosette leaves of *pSTM::STM-VENUS* plants grown under HL. Samples of 35 µg of total protein were loaded from SAM and leaf extracts. Ponceau staining serves as loading control. Similar results were obtained from two independent experiments. (*C*) RT-qPCR analyses of SnRK1 signaling marker genes (*DIN10, SEN5, DRM2*) in SAMs of *pSTM::STM-VENUS* plants grown in HL and transferred to darkness (D) or kept in HL for 48 h. Graphs show the average of three independent samples, each consisting of a pool of five SAMs. Paired ratio *t-*test (*P*-values shown). (*D*) *Left*, representative immunoblot of SnRK1α1 T-loop phosphorylation in SAMs of the plants described in (*C*), using antibodies recognizing the T175 phosphorylation (phospho-SnRK1α) or the total SnRK1α1 protein. *Right*, quantification of the mean SnRK1α phosphorylation (phospho-SnRK1α/total SnRK1α1) in D as compared to the ratio in HL (*n* = 3; each a pool of five SAMs). Paired ratio *t* test (*P*-value shown). (*E* and *F*) STM-VENUS expression in SAMs of control and *SnRK1α1-OE* plants grown under HL or LL conditions (*E*) or grown under HL and transferred to darkness (D) or kept under HL for 48 h (*F*). The same batches of HL-grown plants served as controls for the experiments shown in (*E*) and (*F*). HL and LL-grown STM-VENUS samples are replotted from Fig. 1*B* as a reference. Plots show SAM measurements of plants grown as three independent batches normalized by the mean of the HL condition of each batch (control, HL: *n* = 44, LL: *n* = 45, D: *n* = 45; *SnRK1α1-OE*, HL: *n* = 45, LL: *n* = 45; D: *n* = 45). Different letters indicate statistically significant differences (Kruskal–Wallis with Dunn's test; *P* < 0.05). (*G*) Effect of light on the levels of sugars in SAMs of *SnRK1α1-OE* plants as compared to the control. Suc, sucrose; Tre6P, trehalose 6-phosphate; Glc, glucose; Fru, fructose. Plots show measurements of five to six samples, each consisting of a pool of five SAMs from plants grown as two independent batches. Welch’s *t* test (mutant vs. control for each condition; *P*-values shown). (*H*) Yeast-two hybrid assays examining the interaction of SnRK1α1 with STM. Protein interaction was determined by monitoring yeast growth in medium lacking Leu, Trp, and His (-L-W-H) compared with control medium only lacking Leu and Trp (-L-W). *Upper* panel, yeast growth in cells coexpressing AD-STM, with BD-SnRK1α1. *Lower* panel, negative controls of yeast transfected with the indicated AD/BD-constructs and the complementary BD/AD-empty vectors. BD and AD, DNA binding and activation domains of the GAL4 TF, respectively. Increasing dilutions of transformed yeast cells are shown (10^−1^, 10^−2^, 10^−3^). Experiments were performed three times with similar results. (*I*) Coimmunoprecipitation (co-IP) experiments using *Arabidopsis* Col-0 mesophyll cell protoplasts coexpressing SnRK1α1-HA with STM-GFP or GFP alone. GFP-tagged proteins were immunoprecipitated and coimmunoprecipitation of SnRK1α1 was assessed by immunoblotting with an HA antibody. Arrowheads, STM-GFP (*Upper*) and GFP (*Lower*). Experiments were performed three times with similar results. (*J*) Impact of SnRK1 on STM activity, measured as the induction of the *pCUC1::LUC* reporter in *Arabidopsis* mesophyll protoplasts expressing STM and SnRK1α1 in the indicated combinations. SnRK1α1-KD, kinase-dead SnRK1α1^K48M^ variant. Plots show normalized luciferase (LUC) activity values (*n* = 13 transfections using eight biologically independent protoplast preparations). Different letters denote statistically significant differences (Brown–Forsythe and Welch ANOVA, *P* < 0.05). *Lower* panels, immunoblot analyses of the indicated samples and Ponceau staining of the membrane.

One major component of sugar signaling is the SnRK1 protein kinase, that is activated under conditions of low carbon availability and is conversely repressed by sugars (34). Given its well-established role as a sugar sensor and the increasing number of studies implicating SnRK1 in developmental processes (26, 28), we next investigated whether SnRK1 could be involved in the regulation of SAM function through STM. To this end, we used a line expressing SnRK1α1-GFP under the control of the *SnRK1α1* promoter and other gene regulatory regions (35). A clear SnRK1α1-GFP signal was detected in the SAM, showing a stronger intensity in the peripheral regions, and developing organs (Fig. 3*A*). To further confirm this expression and to assess whether SnRK1α1 might be enriched in the SAM relative to other organs of the plant, we extracted total proteins from rosettes and shoot apices of 6- to 7-wk-old plants and compared the relative levels of SnRK1α1 by immunoblotting (Fig. 3*B*). For the same amount of total protein, shoot apices contained higher amounts of SnRK1α1 suggesting that SnRK1 is relatively more abundant in the SAM than in rosette leaves.

To test whether SnRK1 is functional in the meristem, we used SAMs dissected from HL- or dark-treated plants (48 h) to measure the activity of the SnRK1 signaling pathway using the expression of downstream target genes (34) as readout of in vivo SnRK1 activity (Fig. 3*C*). As expected, SnRK1-regulated starvation genes were barely expressed under control conditions. However, after 48 h of darkness, a marked upregulation of these genes was observed (Fig. 3*C*), indicating an activation of SnRK1 signaling in the SAM. The induction of SnRK1-regulated genes in darkness was accompanied by a reduction in total SnRK1α1 levels (Fig. 3*D*), consistent with a tight coupling between SnRK1 activity and degradation (35, 36), and by an increase in the relative phosphorylation of the SnRK1α1 (T-loop) that is essential for SnRK1 activity (34).

To investigate whether SnRK1 is involved in STM regulation, we used a line overexpressing SnRK1α1 that displays no obvious growth or developmental defects [*35S::SnRK1α1*, hereafter referred to as *SnRK1α1-OE*; (37)]. We introgressed the *pSTM::STM-VENUS* reporter construct into this line and monitored STM-VENUS levels in different light conditions. When plants were grown under HL, the levels of STM-VENUS in *SnRK1α1-OE* were 60% of those in control plants (Fig. 3*E*), a decrease that could not be explained by differences in *STM* transcript levels (*SI Appendix*, Fig. S3). However, the differences between the two genotypes became smaller when plants were grown in LL (STM-VENUS levels in *SnRK1α1-OE* were 77% of those in HL plants; Fig. 3*E*) and negligible when subjected to a 48 h dark treatment (STM-VENUS levels in *SnRK1α1-OE* were 97% of those in control plants; Fig. 3*F*). STM-VENUS levels thus appeared to be constitutively low and largely insensitive to the light conditions in *SnRK1α1-OE* plants. This contrasted with control plants which, in response to restrictive light conditions, reduced STM-VENUS accumulation to levels equivalent to those of *SnRK1α1-OE.* Lower STM-VENUS levels in *SnRK1α1-OE* in HL could not be explained by lower sugar accumulation, as these plants had a higher content of sucrose, glucose, and fructose both in the SAM (Fig. 3*G*) and rosettes (*SI Appendix*, Fig. S4), although the differences were not always statistically significant due to large variation in the *SnRK1α1-OE* samples. The levels of Tre6P, known to inhibit SnRK1 activity (38–40), were also markedly higher in *SnRK1α1-OE* SAMs (5.6-fold) and rosettes (fivefold), consistent with previous observations in *SnRK1α1-OE* rosettes (41). During the dark treatment, however, all sugars were largely depleted, reaching similarly low levels in control and mutant samples.

Altogether these results suggest that SnRK1 is active in the SAM and that it contributes to the adjustment of STM protein levels, inhibiting STM accumulation when sugar levels decline. To further investigate the involvement of SnRK1 on STM regulation, we first used yeast-two-hybrid (Y2H) assays to test whether SnRK1α1 can interact directly with STM (Fig. 3*H*). We observed that yeast coexpressing SnRK1α1 with STM were able to grow in selective medium but this was not the case when SnRK1α1 or STM were expressed individually with the corresponding empty vector controls, suggesting that these two proteins can interact. To determine whether the SnRK1α1-STM interaction can also occur in planta, we next performed coimmunoprecipitation (co-IP) experiments using Col-0 mesophyll cell protoplasts expressing SnRK1α1-HA with STM-GFP or with GFP as a negative control. Immunoprecipitation with an anti-GFP antibody and subsequent immunoblot analyses revealed that SnRK1α1 interacts with STM-GFP (Fig. 3*I*), but not with GFP, indicating that the interactions revealed by Y2H may also occur *in planta*. To explore the functional implications of this interaction, we developed a reporter of STM activity by fusing *LUCIFERASE* (*LUC*) to the promoter of the STM target gene *CUC1* (42) and used the reporter in transient protoplast-based assays (43). STM expression triggered a 17-fold induction of the *CUC1::LUC* reporter and this induction was suppressed by 60% when STM was coexpressed with SnRK1α1, which reduced STM accumulation (Fig. 3*J*). This repressive effect was no longer visible when we used a catalytically inactive SnRK1α1 variant [SnRK1α1^K48M^, (34)], indicating that the impact of SnRK1 on STM depends on the protein-phosphorylating activity of SnRK1. Taken together, our results support that SnRK1α1 regulates STM protein accumulation and activity.

### Silencing *SnRK1α* in the SAM Disrupts Meristem Function.

**Fig. 4.**
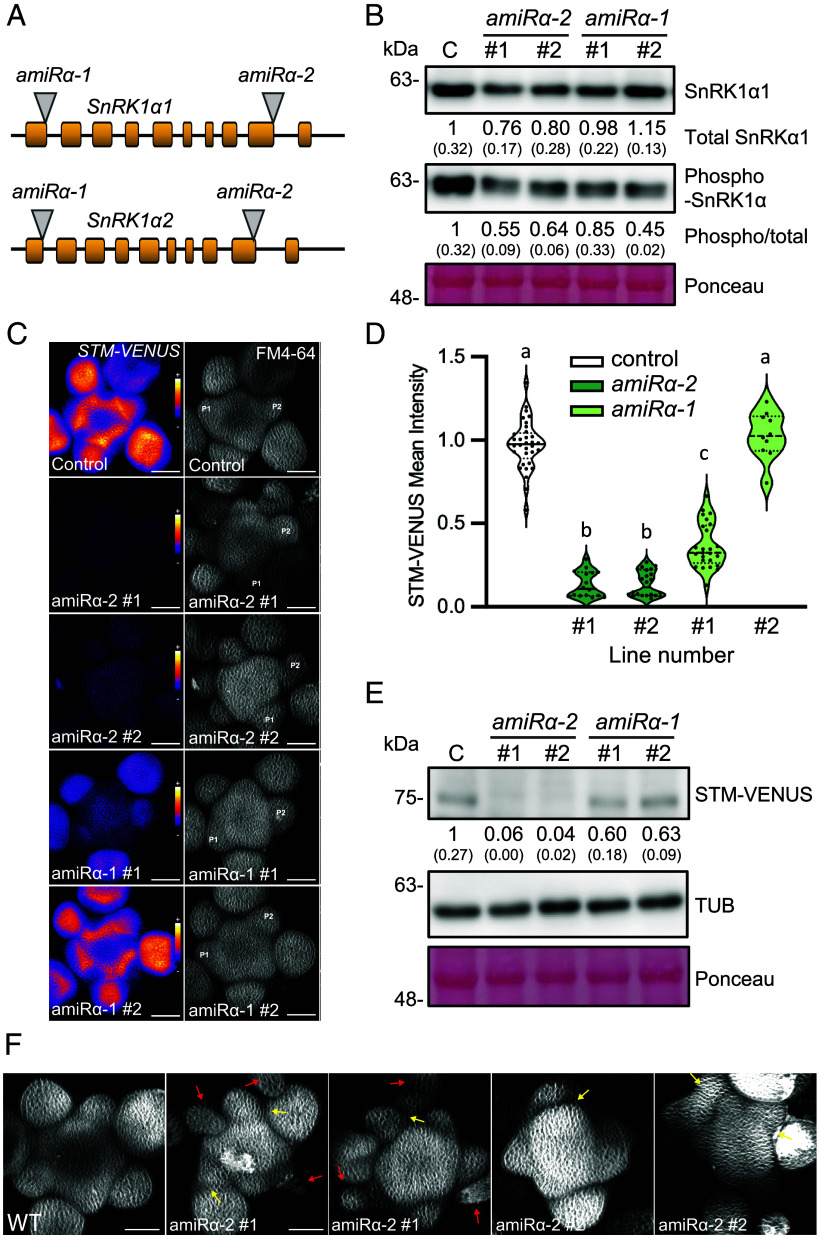
Silencing *SnRK1α* in the SAM leads to reduced STM expression. (*A*) Schematic localization of *amiRα-1* and *amiRα-2* target sites (gray triangles) in the *SnRK1α1* and *SnRK1α2* transcripts. Yellow blocks correspond to exons. (*B*) Immunoblot analyses of SnRK1α T-loop phosphorylation in SAMs of plants expressing *pSTM::amiRα-1* (*amiRα-1*) or *pSTM::amiRα-2* (*amiRα-2*), using antibodies recognizing total SnRK1α1 or SnRK1α phosphorylated on T175 (phospho-SnRK1α). Ponceau staining serves as loading control. Numbers refer to mean SnRK1α1 amounts or mean SnRK1α phosphorylation (phospho-SnRK1α/total SnRK1α1) in the *amiRα* lines relative to the control *STM-VENUS* line (*n* = 2; each a pool of five SAMs; in parentheses, SEM). (*C*) Representative meristems expressing STM-VENUS together with *pSTM::amiRα-1, pSTM::amiRα-2,* or *pSTM::TFP-N7* as a control, and whose membranes were labeled with FM4-64. *Left* panels show the sum-slice projections of the STM-VENUS signal (color-coded using the Fire representation in ImageJ), and the *Right* panels, the sum-slice projection of the FM4-64 signal. (Scale bars, 50 µm.) *P1* and *P2*, youngest visible and older flower primordia, respectively. (*D*) Quantification of the STM-VENUS signal in the SAMs shown in (*C*). Plots show SAM measurements of plants grown as two independent batches (except *amiRα-1#2*, which was grown as a single batch) normalized by the mean of the control line of each batch (control, *n*=34; *amiRα-2#1*, *n*=16; *amiRα-2#2*, *n*=22; *amiRα-1#1*, *n*=25; *amiRα-1#2 n*=10). Different letters indicate statistically significant differences (Kruskal–Wallis with Dunn's test; *P* < 0.05). (*E*) Immunoblot analyses of STM-VENUS and TUBULIN (TUB) protein levels in SAMs of the *amiRα* lines and the *STM-VENUS* control. Ponceau staining serves as loading control. Numbers refer to mean STM-VENUS amounts in the *amiRα* lines as compared to the control (*n* = 2; each a pool of five SAMs; in parentheses, SEM). (*F*) Sum-slice projection of control line and *amiRα-2* showing additional defects in meristem organization. Red arrows point at bract-like structures while yellow arrows point at fusions between adjacent floral primordia. (Scale bars, 50 µm.)

To investigate further the possibility that SnRK1 acts locally in the meristem, we designed artificial microRNAs (amiRNAs) targeting both *SnRK1α1* and *SnRK1α2* in two different regions of the transcripts (*amiRα-1* and *amiRα-2*) and expressed these *amiRNAs* under the 5.7 kb promoter of *STM* in *STM-VENUS* plants (Fig. 4*A*). Immunoblot analyses confirmed a decrease in the activated form (phosphorylated in the T-loop) of SnRK1α in all lines, but this was accompanied by a decrease in total SnRK1α1 levels only in lines expressing *amiRα-2* (Fig. 4*B*).

**Fig. 5.**
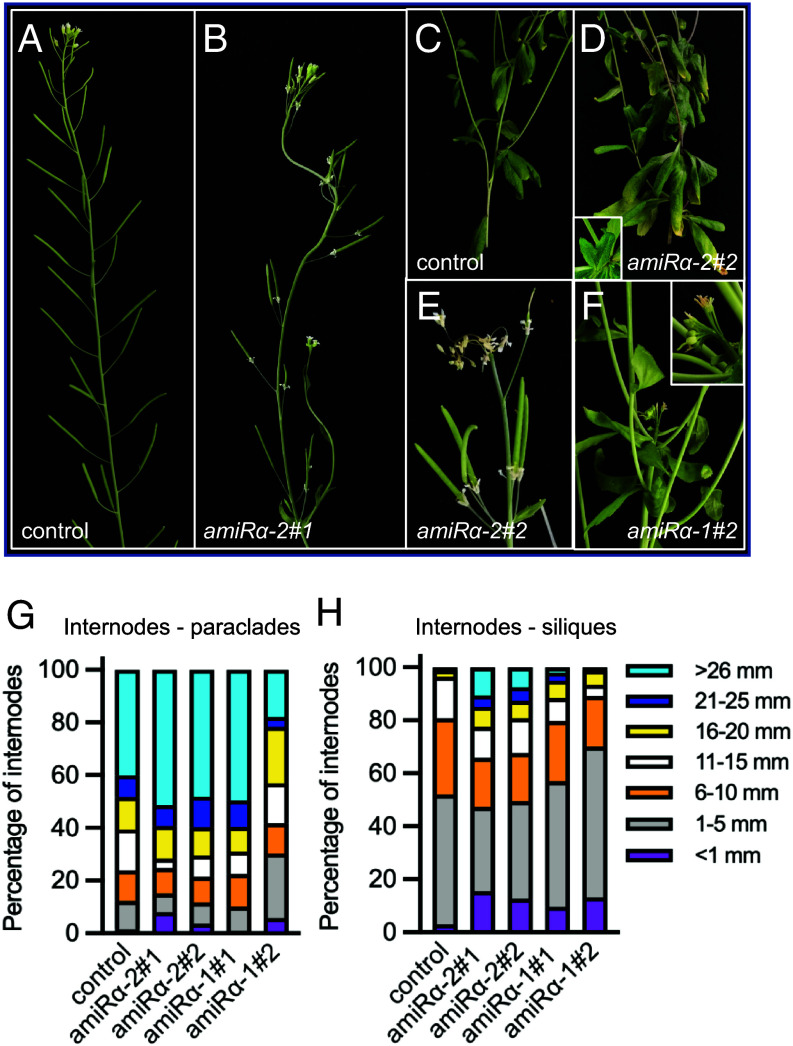
Silencing of *SnRK1α* in the SAM affects meristem function and plant architecture. (*A*–*F*) Representative images of control (*STM-VENUS*, *A* and *C*) and *amiRα* (*B* and *D*–*F*) plants showing irregular internode length (*A* and *B*), clusters of leaves (*C*, *D*, and *F*) and siliques (*A*, *B*, and *E*), and termination of the main inflorescence (*F*) in the *amiRα* lines. *Insets* show organ fusion between leaves of an aerial rosette (*D*) and between pedicels and the stem (*F*). (*G* and *H*) Quantification of the internode length defects in control and two independent lines of *amiRα-1* and *amiRα-2* mutants. Internode length was determined by measuring the length of the internodes between paraclades (*G*) and between the first 12 siliques (*H*), all counted acropetally. Internode length was scored in the indicated size ranges from the main inflorescence of 18 plants of each genotype. Graphs show the relative frequencies of each size class in the total number of internodes scored. All phenotypes were scored from plants grown under equinoctial conditions until the completion of flowering.

To our surprise, SnRK1α depletion resulted in decreased STM-VENUS accumulation in optimal growth conditions (Fig. 4 *C*–*E*) with the signal reaching 14% (*amiRα-2#1* and *amiRα-2#2* lines) and 37% (*amiRα-1#1*) of the control plant levels. This was accompanied by a strong reduction in *STM* transcript levels (*SI Appendix*, Fig. S5), contrasting with the effect of SnRK1α1 overexpression (Fig. 3 *E* and *F* and *SI Appendix*, Fig. S3). The extent of *SnRK1α* depletion correlated with defects in SAM development, including altered phyllotaxy, reduced bulging, and the appearance of bract-like structures in some floral meristems, as well as fusions between adjacent floral meristems (Fig. 4*F*). Organ fusions were also visible later in development and affected cauline and rosette leaves, petals, siliques, and stems (Fig. 5 *D* and *F*). All *amiRα* lines displayed defects in internode elongation, with an increased frequency of aberrantly long and aberrantly short internodes (Fig. 5 *A*, *B*, *G*, and *H*). Defects in internode elongation resulted in clusters of siliques (Fig. 5 *A*, *B*, and *E*) and what appeared to be aerial rosettes on the main inflorescence (Fig. 5 *C*, *D*, and *F*, Table 1, and *SI Appendix*, Fig. S6*A*). These phenotypes were more severe in the *amiRα-2* plants (Fig. 4 *C*–*E*), which also exhibited reduced apical dominance with one or two axillary meristems often becoming activated well before flowering (39% and 28% of the *amiRα-2#1,* and *amiRα-2#2* plants, respectively; *n* = 18; *SI Appendix*, Fig. S6 *B* and *C*). A less frequent termination of the main meristem was also observed, after which growth resumed from an axillary meristem (17% of *amiRα-2#1* plants; *n* = 18; *SI Appendix*, Fig. S6*C*). Plants expressing *pSTM::amiRα* were also compared to the double reporter line as control (*pSTM::STM-VENUS/pSTM:: TFP-N7*) supporting that the observed phenotypes were not caused by the introgression of an additional *STM* promoter in the genome of the *STM-VENUS* line (Fig. 4 *C*, *D*, and *F* and *SI Appendix*, Fig. S6*D*).

**Table 1.** Number of organs per stem node in *amiRα* plants

Line	Siliques per node	*P*-value	Leaves per node	*P*-value
Control	1.4 ± 0.5	—	1.9 ± 0.8	—
*amiRα-2*#1	2.3 ± 0.8	<0.0001	3.8 ± 1.7	<0.0001
*amiRα-2*#2	2.2 ± 0.5	<0.0001	3.6 ± 1.5	<0.0001
*amiRα-1*#1	2.0 ± 0.5	<0.0001	2.8 ± 1.3	<0.0001
*amiRα-1*#2	1.8 ± 0.7	0.0004	2.1 ± 0.9	>0.9999

Measurements were taken from the main inflorescence of 18 plants of the *amiRα* lines and the *STM-VENUS* control line after flowering was completed. Numbers are averages and SD. *P*-values refer to differences between each mutant and the control (Kruskal–Wallis with Dunn's test).

To further examine the consequences of depleting SnRK1 activity in the SAM, we expressed *amiRα-1* and *amiRα-2* under the control of the *RIBOSOMAL PROTEIN 5A* (*RPS5A*) or *FD* promoters, both highly active in inflorescence meristems (44). Three independent lines of each construct were analyzed at the T2 plant stage, revealing phenotypes largely similar to those of the *pSTM::amiRα* lines. All the twelve analyzed lines showed a higher frequency of defective internode elongation than the control, with ten showing also a higher frequency of pedicel fusion (*SI Appendix*, Fig. S7 *A*–*E* and *I*). This was accompanied by a higher incidence of more than one silique per node (*SI Appendix*, Fig. S7*J*). For some of the *pFD::amiRα-1* plants, we also observed severe stem and flower organ fasciation and even meristem abortion (*SI Appendix*, Fig. S7 *F*–*H*).

Collectively, these results indicate that SnRK1 plays critical functions in meristem organization and function.

## Discussion

The capacity to generate organs throughout development is crucial for plant adaptation to the environment. However, how the SAM perceives environmental information and how this is translated into changes in meristem activity are poorly understood.

Here, we show that light promotes STM accumulation through sugars. First, a clear correlation between STM-VENUS and SAM sugar levels was observed across different light conditions. STM-VENUS levels were lower in LL-grown or dark-treated plants than in plants grown and maintained under HL (Fig. 1 *A*–*C*). A similar pattern was observed for sugar accumulation in the inflorescence meristems (Fig. 2*A*), consistent with a previous report on the impact of limiting photosynthetic rates (and thereby sugar supply to the sinks) on the growth and development of reproductive organs and meristem function (45). Second, STM-VENUS levels declined rapidly when inflorescences were excised from rosettes and this decline was similar in inflorescences maintained in the light or transferred to dark, showing that light is not sufficient to sustain STM levels in this system (Fig. 2*B*). The reason for this could be that light is sensed in leaves, generating a systemic light-related signal that is disrupted upon excision of the inflorescence. An alternative explanation is that the signal regulating STM levels is not light itself, but rather photosynthesis-derived sugars. The fact that the decline in STM-VENUS levels triggered by inflorescence excision could be largely suppressed by supplementing sucrose in darkness argues that sucrose is sufficient to sustain STM-VENUS levels and that the effect of light observed in intact plants is indirect via photosynthesis and sugar production. The impact of sucrose on STM is in line with the reported effects of nutrients on *WUS* expression and on meristem function. Sugars contribute to meristem activation by inducing *WUS* in young seedlings (22) and nitrogen promotes *WUS* expression and meristem growth in the inflorescence via systemic CK signaling (14). However, in contrast to *WUS*, for which transcriptional regulation plays a major role (14, 22), we did not detect significant changes in *STM* transcript levels under our different growth conditions or treatments (Fig. 1*E* and *SI Appendix*, Fig. S3), indicating that STM is regulated at the protein level. Despite reports linking CK signaling to *STM* expression (12, 31), STM-VENUS levels did not increase in excised meristems treated with CK (*SI Appendix*, Fig. S1*D*). Even though we did not measure *STM* transcript accumulation under these conditions, this could mean that CK signals may influence STM more indirectly, e.g., by affecting *WUS* expression and stem cell number (3).

The rescue of STM-VENUS levels by sucrose in excised inflorescences suggests that sucrose is sensed locally in the meristem. This is in accordance with the enrichment and activity of the SnRK1 sugar sensor in the SAM (Fig. 3 *A*–*D*). Ubiquitous *SnRK1α1* overexpression caused a reduction in STM-VENUS levels under HL conditions (Fig. 3*E*) despite the high accumulation of soluble sugars and Tre6P in the rosettes and SAMs of the *SnRK1α-OE* plants (Fig. 3*G*). In addition, SnRK1α1 interacted physically with STM in yeast cells and mesophyll cell protoplasts (Fig. 3 *H* and *I*) and repressed STM accumulation and activity in the latter (Fig. 3*J*), suggesting that SnRK1 modulates STM function locally in the meristem.

Depletion of SnRK1 activity via *amiRs* further demonstrated that SnRK1 acts locally in the SAM and that its functions extend beyond STM protein regulation (Figs. 4 and 5 and *SI Appendix*, Figs. S5–S7). Reduced SnRK1 activity caused a wide range of developmental defects which contrasts with the absence of morphological anomalies in the *SnRK1α-OE* line in our and previous studies (26, 28).

The finding that sucrose promotes STM-VENUS protein accumulation together with the fact that sugars repress SnRK1 activity, may at first sight appear to conflict with abnormal meristem function and the decline in *STM* transcript and protein accumulation observed upon *SnRK1α* silencing. However, despite being generally considered a growth repressor, SnRK1 is also required for cell cycle progression (46) and for normal growth and development (47). Indeed, transient *SnRK1α1/α2* down-regulation via virus-induced gene silencing leads to full growth arrest of plants (34) and double *snrk1α1 snrk1α2* null mutants could thus far not be recovered, suggesting that complete loss of SnRK1α is embryo lethal (48). A similar duality is observed for the AMP-activated protein kinase (AMPK), the homologue of SnRK1 in animals. Despite serving as a brake for cell proliferation through downregulation of TOR activity (49), AMPK is also essential for normal growth and development. For example, complete loss of the AMPKß1 subunit leads to cell cycle defects in neural stem and progenitor cells, causing profound abnormalities in brain development in mice (50). Along the same lines, hematopoietic stem cell function in mammals is disrupted both upon inactivation and overactivation of TOR signaling, indicating that a fine balance of this central regulator is required for coordinating cell proliferation, differentiation, and regeneration (51).

The effects of light, sucrose, and SnRK1α1 overexpression on STM indicate that the underlying mechanisms do not rely on changes in *STM* transcript abundance (Fig. 1–3 and *SI Appendix*, Fig. S3). Furthermore, SnRK1α1 and STM proteins interact physically and functionally, and SnRK1α1 kinase activity is needed for the repression of STM function (Fig. 3 *H*–*J*). This suggests that SnRK1 impacts STM protein function, either through phosphorylation of STM or of an STM interactor required for its activity. The consequences of *SnRK1α* silencing, on the other hand, reveal a more complex scenario, causing severe developmental abnormalities that are accompanied by a reduction in the *STM* transcript and a more pronounced reduction in STM protein accumulation (Figs. 4 and 5 and *SI Appendix*, Figs. S5–S7). In this case, the impact of SnRK1α on STM is likely to be largely indirect, involving processes (e.g., hormone signaling, the cell cycle, others) that remain to be determined.

**Fig. 6.**
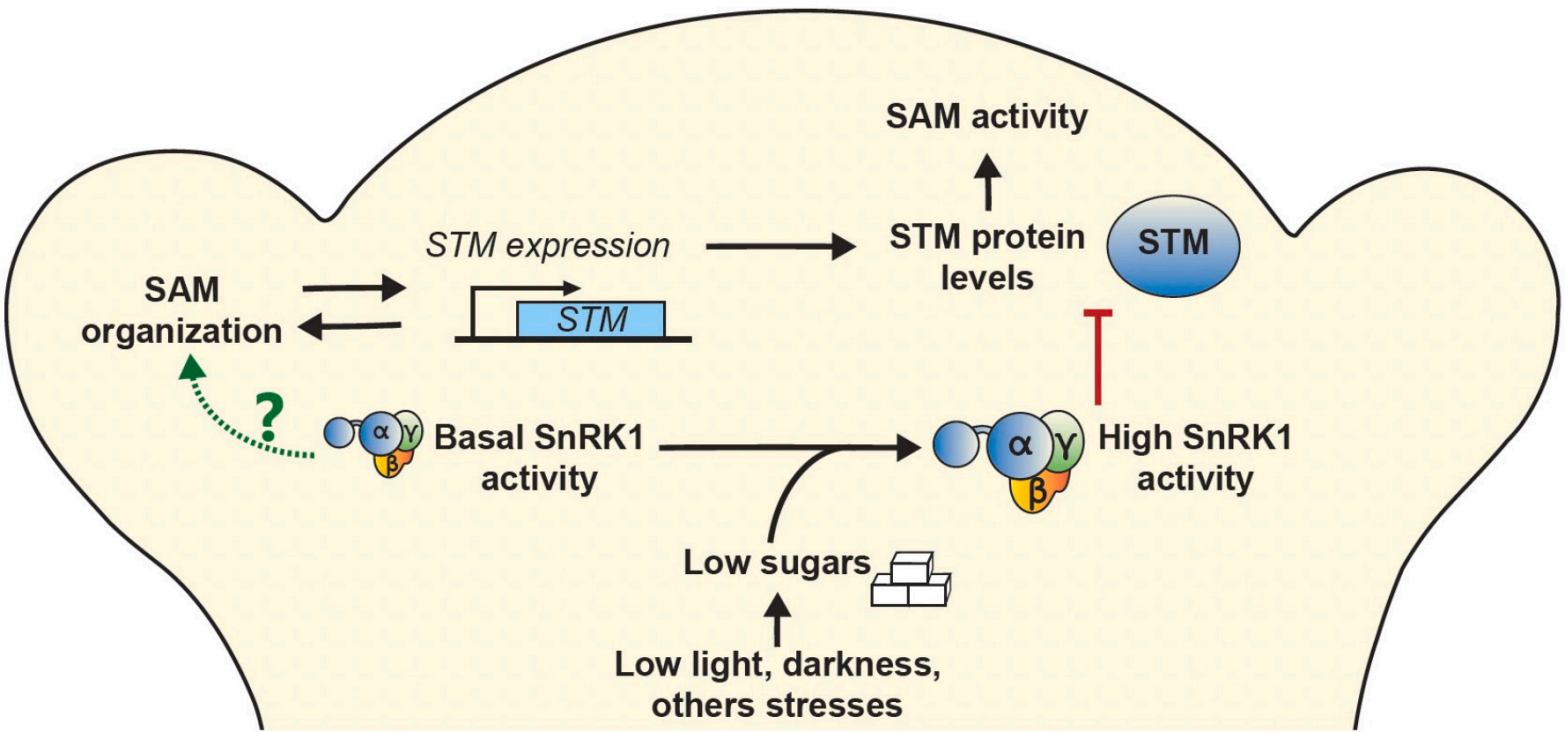
Model for the role of sugars and SnRK1 signaling in the SAM. *Left*, under favorable conditions, basal SnRK1 activity is required for meristem organization, with local *SnRK1α* silencing causing severe phenotypes related to SAM dysfunction and, likely as a consequence, reduced *STM* expression. The mechanisms underlying these SnRK1 effects remain unknown (indicated by a question mark). *Right*, under limiting light conditions or other unfavorable situations, sugar levels decrease, leading to a strong activation of SnRK1 signaling. This results in decreased STM protein accumulation, potentially through direct action of SnRK1α1 on STM or an STM partner to reduce SAM activity and growth.

Altogether, our work demonstrates that sucrose promotes STM accumulation and that this is counteracted by the SnRK1 sugar sensor, likely to adjust SAM activity to the environment (Fig. 6). Nevertheless, SnRK1 is also essential for the maintenance of meristem functions in optimal growth conditions, adding to the evidence that SnRK1 performs a dual function in the regulation of growth and that its activity needs to be finely balanced.

## Materials and Methods

A list of all primers, plant lines, and antibodies used in this study is provided in *SI Appendix*. Details of plant growth and treatment conditions, protein extraction and quantification, immunoblotting, RNA extraction, cDNA synthesis, qRT-PCR, sugar measurements, yeast-two-hybrid assays, and protoplast assays are described in *SI Appendix*.

### Plant Material.

All *Arabidopsis thaliana* (L.) Heynh. plants used here are in the Columbia (Col-0) background. The *pSTM::STM-VENUS* line (*STM-VENUS*) was generated by transforming Col-0 plants with the plasmid described by Heisler et al. (14, 29). The *pTCSn::GFP* line was provided by Bruno Müller (32). The *SnRK1α1-GFP* [*pSnRK1α1::SnRK1α1-GFP::terSnRK1α1/snrk1α1-3*; (35)] and *SnRK1α1-OE* [*35S::SnRK1α1*; (37)] lines were previously described. For expression of STM-VENUS in the *SnRK1α1-OE* background, the *SnRK1α1-OE* and *STM-VENUS* lines were crossed, and homozygous progeny was selected on kanamycin and BASTA. For generating the *pSTM::STM-VENUS/pSTM::TFP-N7* line and the *pSTM::amiRα* lines, *STM-VENUS* plants were transformed with a construct to express TFP-N7 or an amiRNA targeting both *SnRK1α1* and *SnRK1α2* (*amiRα-1* or *amiRα-2*) under the *STM* promoter (5.7 kb). For generating the *pRPS5a::amiRα* and *pFD::amiRα* lines, *SnRK1α1-GFP* plants were transformed with a construct to express *amiRα-1* or *amiRα-2* under the *RPS5a* (1.7 kb) or *FD* (3 kb) promoters. Detailed descriptions of the cloning strategy and progeny selection are provided in *SI Appendix*.

### SAM Imaging and Quantification.

For meristem imaging, the main inflorescence meristem of plants at the beginning of the flowering stage was cut 1 to 2 cm from the tip, dissected under a binocular stereoscopic microscope to remove all the flowers down to stage 3 [as defined in ref. 52] and transferred to a box containing *Arabidopsis* apex culture medium without sucrose [ACM: 2.2 g/L Duchefa Biochemie (https://www.duchefa-biochemie.com/)-MS basal salt mixture with vitamins, pH adjusted to 5.8 with KOH, and 1.6% (w/v) agarose added].

For the time-lapse experiments examining the effect of exogenous CK, meristems were dissected from HL-grown plants and placed in a box of ACM with 1% (w/v) sucrose and 500 nM BAP or the equivalent volume of the BAP solvent (DMSO) as control. Meristems were thereafter returned to the constant HL cabinet for the indicated times and covered with water for imaging.

For the experiments examining the effect of exogenous sugar, inflorescences were dissected at about 3 cm from the apex from HL-grown plants at the beginning of the flowering stage and placed in a 2 mL Eppendorf tube containing 2 mL of liquid ACM (no sucrose) covered with parafilm pierced with a needle so that the inflorescence could be held in air while the base of the stem was submerged in the solution, supplemented with the indicated concentrations of sucrose or sorbitol as osmotic control. Sorbitol and sucrose were used at near isosmotic concentrations, with 1% (w/v) sorbitol and 2% (w/v) sucrose corresponding to 54 mM and 58 mM, respectively. Inflorescences were thereafter kept in darkness inside the growth cabinet for the indicated times. Meristems were then dissected from the excised inflorescences, transferred to a box containing the same sucrose- or sorbitol-supplemented solid [1.6% (w/v) agarose)] medium and covered with water for imaging.

## Supplementary Material

Appendix 01 (PDF)

## Data Availability

All study data are included in the article and/or *SI Appendix*.

## References

[1] M. K. Barton, Twenty years on: The inner workings of the shoot apical meristem, a developmental dynamo. Dev. Biol. **341**, 95–113 (2010). 19961843 10.1016/j.ydbio.2009.11.029

[2] M. Galli, A. Gallavotti, Expanding the regulatory network for meristem size in plants. Trends Genet. **32**, 372–383 (2016). 27129984 10.1016/j.tig.2016.04.001

[3] F. L. Lopes, C. Galvan-Ampudia, B. Landrein, WUSCHEL in the shoot apical meristem: Old player, new tricks. J. Exp. Bot. **72**, 1527–1535 (2021). 33332559 10.1093/jxb/eraa572

[4] J. A. Long, E. I. Moan, J. I. Medford, M. K. Barton, A member of the KNOTTED class of homeodomain proteins encoded by the STM gene of *Arabidopsis*. Nature **379**, 66–69 (1996). 8538741 10.1038/379066a0

[5] M. K. Barton, R. S. Poethig, Formation of the shoot apical meristem in *Arabidopsis thaliana*: An analysis of development in the wild type and in the shoot meristemless mutant. Development **119**, 823–831 (1993).

[6] K. Endrizzi, B. Moussian, A. Haecker, J. Z. Levin, T. Laux, The SHOOT MERISTEMLESS gene is required for maintenance of undifferentiated cells in *Arabidopsis* shoot and floral meristems and acts at a different regulatory level than the meristem genes WUSCHEL and ZWILLE. Plant J. **10**, 967–979 (1996). 9011081 10.1046/j.1365-313x.1996.10060967.x

[7] S. E. Clark, S. E. Jacobsen, J. Z. Levin, E. M. Meyerowitz, The CLAVATA and SHOOT MERISTEMLESS loci competitively regulate meristem activity in *Arabidopsis*. Development **122**, 1567–1575 (1996). 8625843 10.1242/dev.122.5.1567

[8] M. E. Byrne , Asymmetric leaves1 mediates leaf patterning and stem cell function in *Arabidopsis*. Nature **408**, 967–971 (2000). 11140682 10.1038/35050091

[9] S. Scofield, W. Dewitte, J. Nieuwland, J. A. H. Murray, The *Arabidopsis* homeobox gene *SHOOT MERISTEMLESS* has cellular and meristem-organisational roles with differential requirements for cytokinin and CYCD3 activity. Plant J. **75**, 53–66 (2013). 23573875 10.1111/tpj.12198

[10] S. Scofield , Coordination of meristem and boundary functions by transcription factors in the SHOOT MERISTEMLESS regulatory network. Development **145**, dev157081 (2018). 29650590 10.1242/dev.157081 PMC5992597

[11] O. Yanai , *Arabidopsis* KNOXI proteins activate cytokinin biosynthesis. Curr. Biol. **15**, 1566–1571 (2005). 16139212 10.1016/j.cub.2005.07.060

[12] H.-M. Rupp, M. Frank, T. Werner, M. Strnad, T. Schmulling, Increased steady state mRNA levels of the STM and KNAT1 homeobox genes in cytokinin overproducing *Arabidopsis thaliana* indicate a role for cytokinins in the shoot apical meristem. Plant J. **18**, 557–563 (1999). 10417706 10.1046/j.1365-313x.1999.00472.x

[13] S. P. Gordon, V. S. Chickarmane, C. Ohno, E. M. Meyerowitz, Multiple feedback loops through cytokinin signaling control stem cell number within the *Arabidopsis* shoot meristem. Proc. Natl. Acad. Sci. U.S.A. **106**, 16529–16534 (2009). 19717465 10.1073/pnas.0908122106 PMC2752578

[14] B. Landrein , Nitrate modulates stem cell dynamics in *Arabidopsis* shoot meristems through cytokinins. Proc. Natl. Acad. Sci. U.S.A. **115**, 1382–1387 (2018). 29363596 10.1073/pnas.1718670115 PMC5819446

[15] Y. H. Su , Integration of pluripotency pathways regulates stem cell maintenance in the *Arabidopsis* shoot meristem. Proc. Natl. Acad. Sci. U.S.A. **117**, 22561–22571 (2020). 32839309 10.1073/pnas.2015248117 PMC7486707

[16] O. Hamant, V. Pautot, Plant development: A TALE story. C. R. Biol. **333**, 371–381 (2010). 20371112 10.1016/j.crvi.2010.01.015

[17] M. Cole, C. Nolte, W. Werr, Nuclear import of the transcription factor SHOOT MERISTEMLESS depends on heterodimerization with BLH proteins expressed in discrete sub-domains of the shoot apical meristem of *Arabidopsis thaliana*. Nucleic Acids Res. **34**, 1281–1292 (2006). 16513846 10.1093/nar/gkl016 PMC1388269

[18] S. Kimura, D. Koenig, J. Kang, F. Y. Yoong, N. Sinha, Natural variation in leaf morphology results from mutation of a novel KNOX gene. Curr. Biol. **18**, 672–677 (2008). 18424140 10.1016/j.cub.2008.04.008

[19] B. Rutjens , Shoot apical meristem function in *Arabidopsis* requires the combined activities of three BEL1-like homeodomain proteins. Plant J. **58**, 641–654 (2009). 19175771 10.1111/j.1365-313X.2009.03809.x

[20] S. Yoshida, T. Mandel, C. Kuhlemeier, Stem cell activation by light guides plant organogenesis. Genes Dev. **25**, 1439–1450 (2011). 21724835 10.1101/gad.631211 PMC3134086

[21] A. Pfeiffer, C. Wenzl, J. U. Lohmann, Beyond flexibility: Controlling stem cells in an ever changing environment. Curr. Opin. Plant Biol. **35**, 117–123 (2017). 27918940 10.1016/j.pbi.2016.11.014

[22] A. Pfeiffer , Integration of light and metabolic signals for stem cell activation at the shoot apical meristem. eLife **5**, e17023 (2016). 27400267 10.7554/eLife.17023 PMC4969040

[23] X. Li , Differential TOR activation and cell proliferation in *Arabidopsis* root and shoot apexes. Proc. Natl. Acad. Sci. U.S.A. **114**, 2765–2770 (2017). 28223530 10.1073/pnas.1618782114 PMC5347562

[24] Y. Xiong , Glucose–TOR signalling reprograms the transcriptome and activates meristems. Nature **496**, 181–186 (2013). 23542588 10.1038/nature12030 PMC4140196

[25] D. Janocha , TOR kinase controls shoot development by translational regulation of cytokinin catabolic enzymes. bioRxiv [Preprint] (2021). 10.1101/2021.07.29.454319 (Accessed 20 November 2022).

[26] E. Baena-González, J. Hanson, Shaping plant development through the SnRK1–TOR metabolic regulators. Curr. Opin. Plant Biol. **35**, 152–157 (2017). 28027512 10.1016/j.pbi.2016.12.004

[27] L. Margalha, A. Confraria, E. Baena-González, SnRK1 and TOR: Modulating growth–defense trade-offs in plant stress responses. J. Exp. Bot. **70**, 2261–2274 (2019). 30793201 10.1093/jxb/erz066

[28] M. Jamsheer, M. Kumar, V. Srivastava, SNF1-related protein kinase 1: The many-faced signaling hub regulating developmental plasticity in plants. J. Exp. Bot. **72**, 6042–6065 (2021). 33693699 10.1093/jxb/erab079

[29] M. G. Heisler , Patterns of auxin transport and gene expression during primordium development revealed by live imaging of the *Arabidopsis* inflorescence meristem. Curr. Biol. **15**, 1899–1911 (2005). 16271866 10.1016/j.cub.2005.09.052

[30] B. Landrein , Mechanical stress contributes to the expression of the STM homeobox gene in *Arabidopsis* shoot meristems. eLife **4**, e07811 (2015). 26623515 10.7554/eLife.07811 PMC4666715

[31] W. L. L. Teo, P. Kumar, C. J. Goh, S. Swarup, The expression of Brostm, a KNOTTED1-like gene, marks the cell type and timing of in vitro shoot induction in *Brassica oleracea*. Plant Mol. Biol. **46**, 567–580 (2001). 11516150 10.1023/a:1010686931889

[32] E. Zürcher , A robust and sensitive synthetic sensor to monitor the transcriptional output of the cytokinin signaling network in planta. Plant Physiol. **161**, 1066–1075 (2013). 23355633 10.1104/pp.112.211763 PMC3585579

[33] F. Fichtner, J. E. Lunn, The role of trehalose 6-phosphate (Tre6P) in plant metabolism and development. Annu. Rev. Plant Biol. **72**, 737–760 (2021). 33428475 10.1146/annurev-arplant-050718-095929

[34] E. Baena-González, F. Rolland, J. M. Thevelein, J. Sheen, A central integrator of transcription networks in plant stress and energy signalling. Nature **448**, 938–942 (2007). 17671505 10.1038/nature06069

[35] P. Crozet , SUMOylation represses SnRK1 signaling in *Arabidopsis*. Plant J. **85**, 120–133 (2016). 26662259 10.1111/tpj.13096 PMC4817235

[36] J. Sun , GRIK phosphorylates and activates KIN10 which also promotes its degradation. Front. Plant Sci. **15**, 1375471 (2024). 38590740 10.3389/fpls.2024.1375471 PMC10999582

[37] M. Jossier , SnRK1 (SNF1-related kinase 1) has a central role in sugar and ABA signalling in *Arabidopsis thaliana*. Plant J. **59**, 316–328 (2009). 19302419 10.1111/j.1365-313X.2009.03871.x

[38] Y. Zhang , Inhibition of SNF1-related protein kinasel activity and regulation of metabolic pathways by trehalose-6-phosphate1. Plant Physiol. **149**, 1860–1871 (2009). 19193861 10.1104/pp.108.133934 PMC2663748

[39] Z. Zhai , Trehalose 6-phosphate positively regulates fatty acid synthesis by stabilizing wrinkled1. Plant Cell **30**, 2616–2627 (2018). 30249634 10.1105/tpc.18.00521 PMC6241258

[40] C. Nunes , Inhibition of SnRK1 by metabolites: Tissue-dependent effects and cooperative inhibition by glucose 1-phosphate in combination with trehalose 6-phosphate. Plant Physiol. Biochem. **63**, 89–98 (2013). 23257075 10.1016/j.plaphy.2012.11.011

[41] B. Peixoto , Impact of the SnRK1 protein kinase on sucrose homeostasis and the transcriptome during the diel cycle. Plant Physiol. **187**, 1357–1373 (2021). 34618060 10.1093/plphys/kiab350 PMC8566312

[42] S. V. Spinelli, A. P. Martin, I. L. Viola, D. H. Gonzalez, J. F. Palatnik, A mechanistic link between *STM* and *CUC1* during *Arabidopsis* development. Plant Physiol. **156**, 1894–1904 (2011). 21685178 10.1104/pp.111.177709 PMC3149926

[43] S.-D. Yoo, Y.-H. Cho, J. Sheen, *Arabidopsis* mesophyll protoplasts: A versatile cell system for transient gene expression analysis. Nat. Protoc. **2**, 1565–1572 (2007). 17585298 10.1038/nprot.2007.199

[44] J. Waese , ePlant: Visualizing and exploring multiple levels of data for hypothesis generation in plant biology. Plant Cell **29**, 1806–1821 (2017). 28808136 10.1105/tpc.17.00073 PMC5590499

[45] M. A. Lauxmann , Reproductive failure in *Arabidopsis thaliana* under transient carbohydrate limitation: Flowers and very young siliques are jettisoned and the meristem is maintained to allow successful resumption of reproductive growth. Plant Cell Environ. **39**, 745–767 (2016). 26351840 10.1111/pce.12634

[46] T. Guérinier , Phosphorylation of p27(^KIP1^) homologs KRP6 and 7 by SNF1-related protein kinase-1 links plant energy homeostasis and cell proliferation. Plant J. **75**, 515–525 (2013). 23617622 10.1111/tpj.12218

[47] E. Baena-González, J. E. Lunn, SnRK1 and trehalose 6-phosphate—two ancient pathways converge to regulate plant metabolism and growth. Curr. Opin. Plant Biol. **55**, 52–59 (2020). 32259743 10.1016/j.pbi.2020.01.010

[48] M. Ramon , Default activation and nuclear translocation of the plant cellular energy sensor SnRK1 regulate metabolic stress responses and development. Plant Cell **31**, 1614–1632 (2019). 31123051 10.1105/tpc.18.00500 PMC6635846

[49] A. González, M. N. Hall, S. C. Lin, D. G. Hardie, AMPK and TOR: The Yin and Yang of cellular nutrient sensing and growth control. Cell Metab. **31**, 472–492 (2020). 32130880 10.1016/j.cmet.2020.01.015

[50] B. Dasgupta, J. Milbrandt, AMP-activated protein kinase phosphorylates retinoblastoma protein to control mammalian brain development. Dev. Cell **16**, 256–270 (2009). 19217427 10.1016/j.devcel.2009.01.005 PMC2662481

[51] D. Kalaitzidis , mTOR complex 1 plays critical roles in hematopoiesis and Pten-loss-evoked leukemogenesis. Cell Stem Cell **11**, 429–439 (2012). 22958934 10.1016/j.stem.2012.06.009 PMC3743253

[52] D. R. Smyth, J. L. Bowman, E. M. Meyerowitz, Early flower development in *Arabidopsis*. Plant Cell **2**, 755–767 (1990). 2152125 10.1105/tpc.2.8.755 PMC159928

