# Supplementary material for: Sugar signaling modulates SHOOT MERISTEMLESS expression and meristem function in Arabidopsis
Source: Proc Natl Acad Sci U S A. 2024 Sep 6;121(37):e2408699121. doi: 10.1073/pnas.2408699121 (PMC11406306; doi:10.1073/pnas.2408699121)
Supplement: Supplementary file 1 — Appendix 01 (PDF) [file pnas.2408699121.sapp.pdf]

Supplementary Figure S1

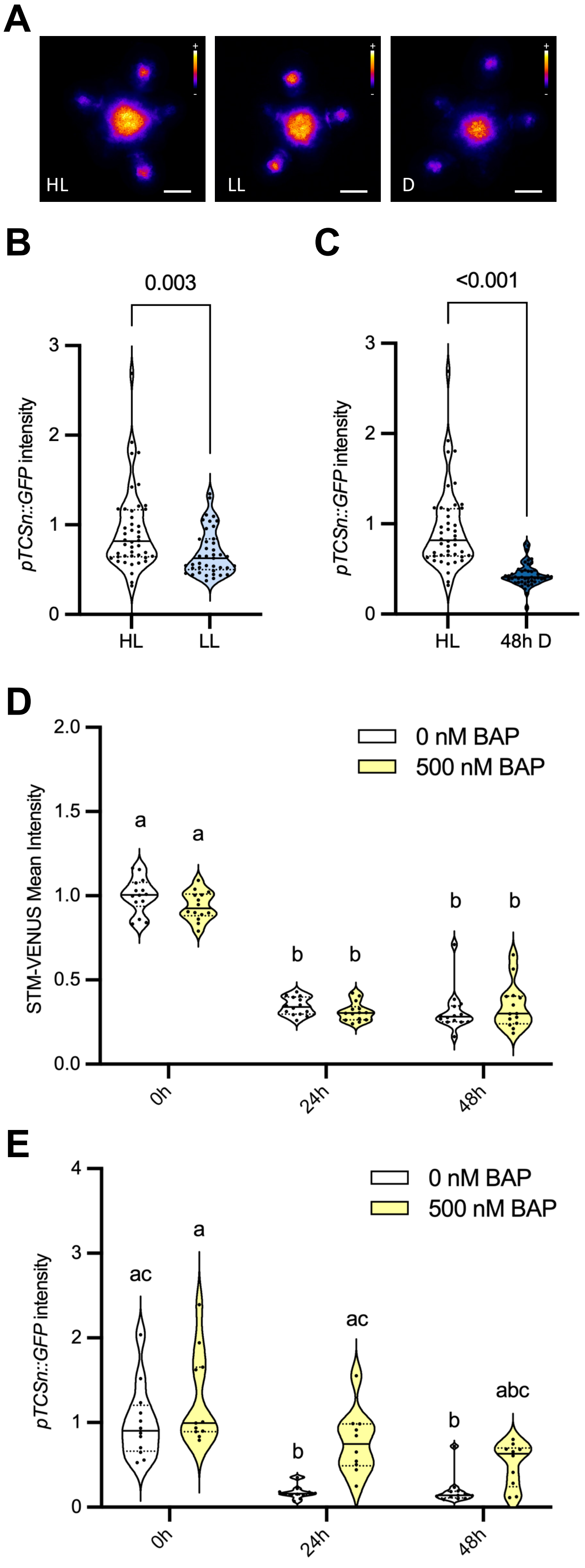

Supplementary Figure S1. Effect of cytokinin on STM levels. A, Representative GFP images from *pTCSn::GFP* SAMs from plants grown under high light (HL; 170 µmol m^{-2} s^{-1}) or low light (LL; 60 µmol m^{-2} s^{-1}) conditions and of plants transferred from HL to darkness (D) for 48 h. Scale bar, 50 µm. **B,** GFP quantification from SAMs of *pTCSn::GFP* plants grown under HL or LL conditions. Plots show SAM measurements of plants grown as three independent batches normalized by the mean of the HL condition of each batch (HL: n=44, LL: n=41). Student's t-test (p-values shown). **C,** GFP quantification from SAMs of *pTCSn::GFP* plants grown in HL and transferred to D or kept under HL for 48 h. Plots show SAM measurements of plants grown as three independent batches normalized by the mean of the HL condition of each batch (HL, n=44; 48 h D, n=45). Student's t-test (p-values shown). **D,** Effect of cytokinin (BAP) application on the expression of the STM-VENUS reporter. SAMs of plants grown under HL conditions were excised and placed in medium supplemented or not with 500 nM BAP for the indicated times. Plots show SAM measurements of plants grown as two independent batches normalized by the mean of the uncut (0 h) condition of each batch (n=14 for each of the indicated conditions). Different letters indicate statistically significant differences (Kruskal-Wallis with Dunn´s test; p<0.05). **E,** Effect of cytokinin (BAP) application on the activity of the *pTCSn::GFP* reporter. SAMs of plants grown under HL conditions were excised and placed in medium supplemented or not with 500 nM BAP for the indicated times. Plots show SAM measurements of plants grown as two independent batches normalized by the mean of the uncut (0h) condition of each batch (all 0 nM BAP conditions, n=12; 0 h 500 nM BAP, n=12; 24 h 500 nM BAP, n=10; 48 h 500 nM BAP, n=10). Different letters indicate statistically significant differences (Kruskal-Wallis with Dunn´s test; p<0.05).

Supplementary Figure S2

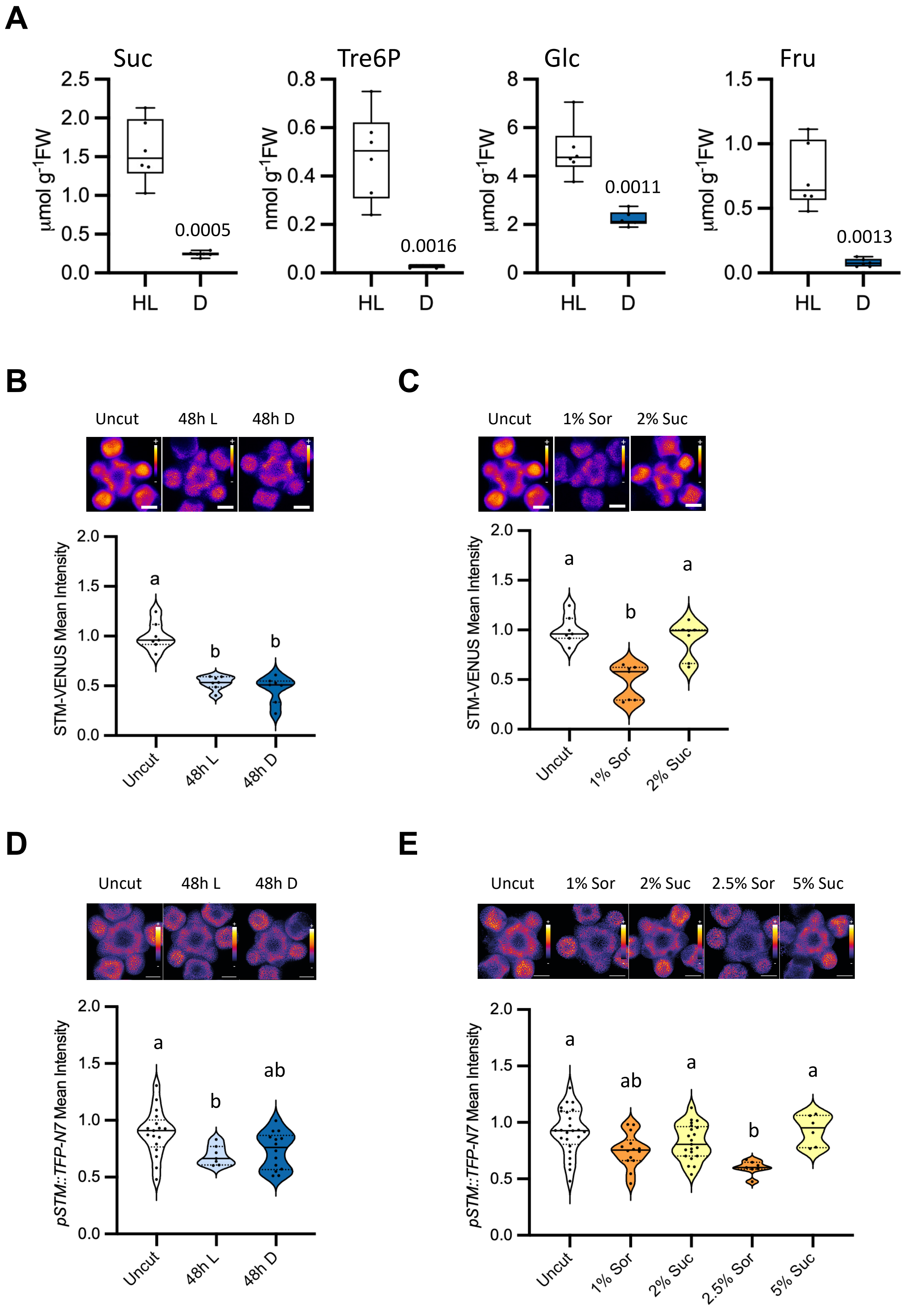

Supplementary Figure S2. Effect of sugar on *STM* promoter activity. A, Effect of light on the levels of soluble sugars in rosettes of *pSTM::STM-VENUS* plants grown in high light (HL) and transferred to darkness (D) or kept in HL for 48 h. Suc, sucrose; Tre6P, trehalose 6-phosphate; Glc, glucose; Fru, fructose. Plots show measurements of six whole rosettes from plants grown as one batch. Welch´s *t*-test (*p*-value shown). **B,** Effect of light on STM-VENUS levels in cut inflorescences. Inflorescences of *pSTM::STM-VENUS* plants grown under HL were cut and placed in medium without sugar for 48 h under HL (L) or dark (D) conditions, after which the SAMs were dissected and imaged (VENUS). Upper panel, representative STM-VENUS images of SAMs. Scale bar, 50 µm. Lower panel, plots showing SAM measurements of plants grown as one batch normalized by the mean of the uncut condition (uncut, *n*=7; 48 h L, *n*=7; 48 h D, *n*=7). Different letters indicate statistically significant differences (Kruskal-Wallis with Dunn´s test; $p<0.05$). **C,** Effect of sugar on STM-VENUS levels in cut inflorescences. Inflorescences of *pSTM::STM-VENUS* plants grown under HL condition were cut and placed under darkness for 48 h in medium with 2% sucrose or 1% sorbitol as osmotic control. SAMs were thereafter dissected and imaged (VENUS). Upper panel, representative STM-VENUS images of SAMs. Scale bar, 50 µm. Lower panel, plots showing SAM measurements of plants grown as one batch normalized by the mean of the uncut condition (uncut, *n*=7; 1% Sor, *n*=7; 2% Suc, *n*=7). Different letters indicate statistically significant differences (Kruskal-Wallis with Dunn´s test; $p<0.05$). The same batches of HL-grown uncut plants served as controls for the experiments shown in (**B**) and (**C**). **D,** Effect of light on *STM* promoter activity in cut inflorescences. Inflorescences of *pSTM::STM-VENUS/pSTM::TFP-N7* plants grown under HL were treated as in (B) and dissected SAMs were imaged (TFP). Upper panel, representative *pSTM::TFP-N7* images of SAMs. Scale bar, 50 µm. Lower panel, plots showing SAM measurements of plants grown as two independent batches (except 48h L samples, which originated from a single batch) normalized by the mean of the *uncut* condition of each batch (uncut, *n*=17; 48 h L, *n*=7; 48 h D, *n*=14). Different letters indicate statistically significant differences (Kruskal-Wallis with Dunn´s test; $p<0.05$). **E,** Effect of sugar on *STM* promoter activity in cut inflorescences. Inflorescences of *pSTM::STM-VENUS/pSTM::TFP-N7* plants grown under HL were cut and placed under darkness for 48 h in medium with sucrose (Suc; 2% and 5%) or sorbitol (Sor; 1% and 2.5%) as osmotic control. SAMs were thereafter dissected and imaged (TFP). Upper panel, representative *pSTM::TFP-N7* images of SAMs. Scale bar, 50 µm. Lower panel, plots showing SAM measurements of plants grown as two to three independent batches (except 2.5% Sor and 5% Suc, which were grown as a single batch) normalized by the mean of the *uncut* condition of each batch (uncut, *n*=24; 1% Sor, *n*=14; 2% Suc, *n*=21; 2.5% Sor, *n*=7; 5% Suc, *n*=6). Different letters indicate statistically significant differences (Kruskal-Wallis with Dunn´s test; $p<0.05$).

Supplementary Figure S3

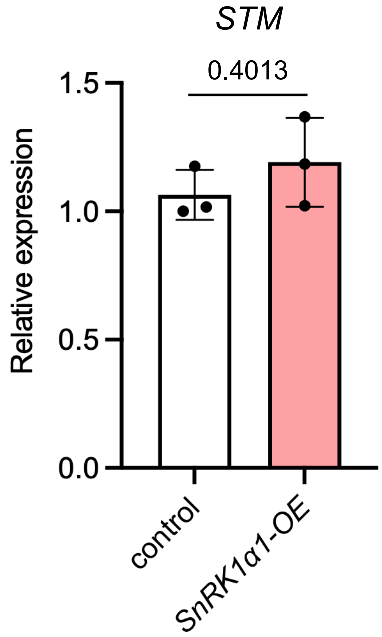

Supplementary Figure S3. Effect of ubiquitous SnRK1α1 overexpression on *STM* levels. RT-qPCR analyses of *STM* in SAMs of control and *SnRK1α1-OE* plants grown under high light conditions (170 µmol m^{-2} s^{-1}). These correspond to the same HL samples shown in Fig. 3E-F. Graphs correspond to the average of three independent samples, each consisting of a pool of five SAMs. Paired ratio *t*-test (*p*-value shown).

Supplementary Figure S4

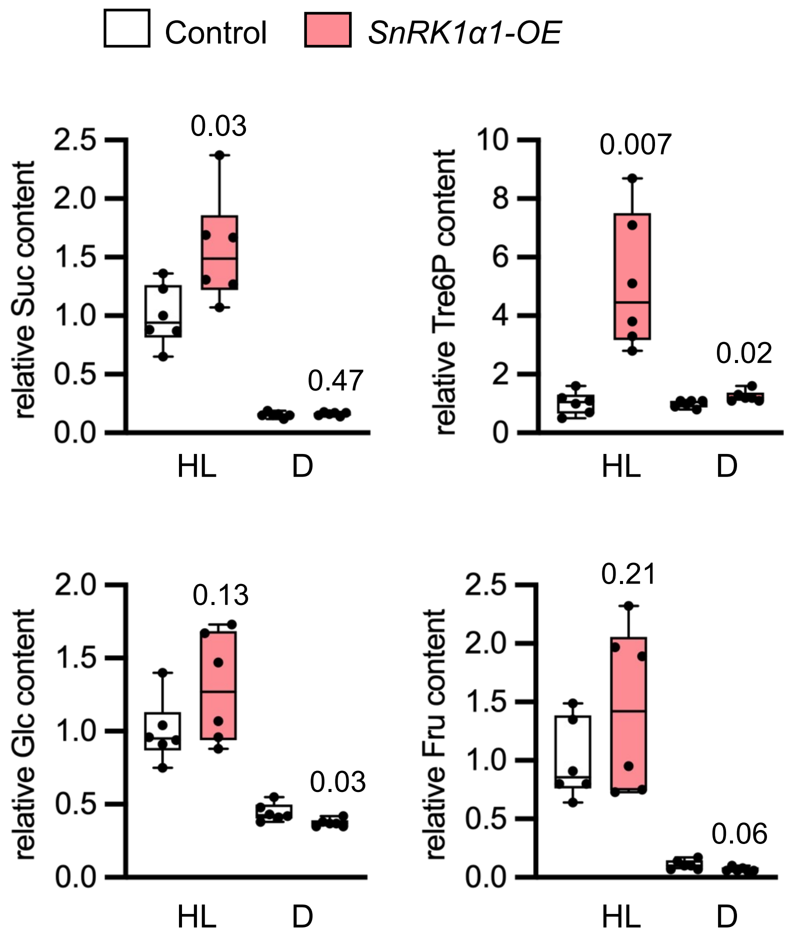

Supplementary Figure S4. Effect of ubiquitous SnRK1α1 overexpression on the accumulation of soluble sugars. Control and *SnRK1α1-OE* plants were grown under HL conditions and transferred to darkness (D) or kept under HL for 48 h. Plots show measurements of six whole rosettes from plants grown as one batch, with *SnRK1α1-OE* values expressed in comparison to the control. Suc, sucrose; Tre6P, trehalose 6-phosphate. Glc, glucose; Fru, fructose. Welch´s *t*-test (mutant *vs*. control for each condition; *p*-value shown).

Supplementary Figure S5

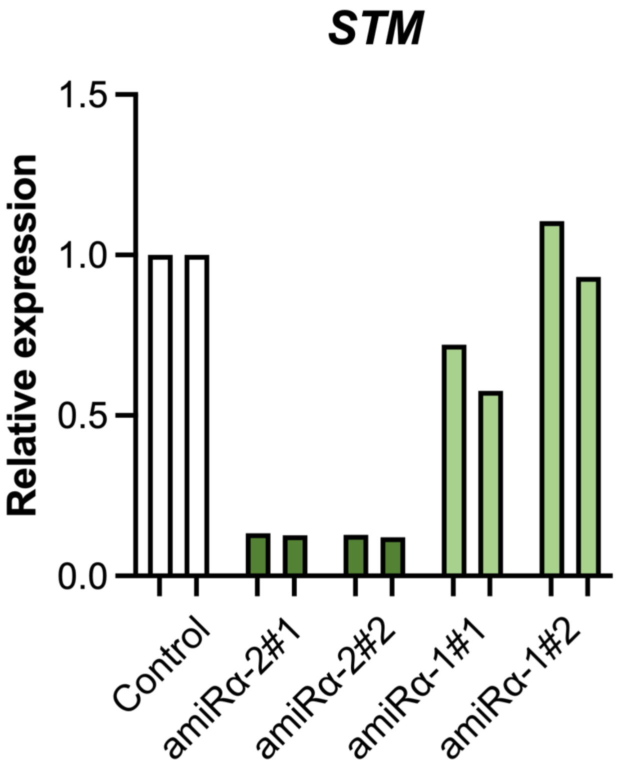

Supplementary Figure S5. Effect of SnRK1α depletion in the SAM on *STM* levels. RT-qPCR analyses of STM in SAMs of control and *pSTM::amiRα* plants grown under high light conditions (170 µmol m^{-2} s^{-1}). Two independent lines of *pSTM::amiRα-2* (*amiRα-2*#1 and *amiRα-2*#2) and *pSTM::amiRα-1* (*amiRα-1*#1 and *amiRα-1*#2) were used. Graph shows measurements from two independent samples, each consisting of a pool of five SAMs.

Supplementary Figure S6

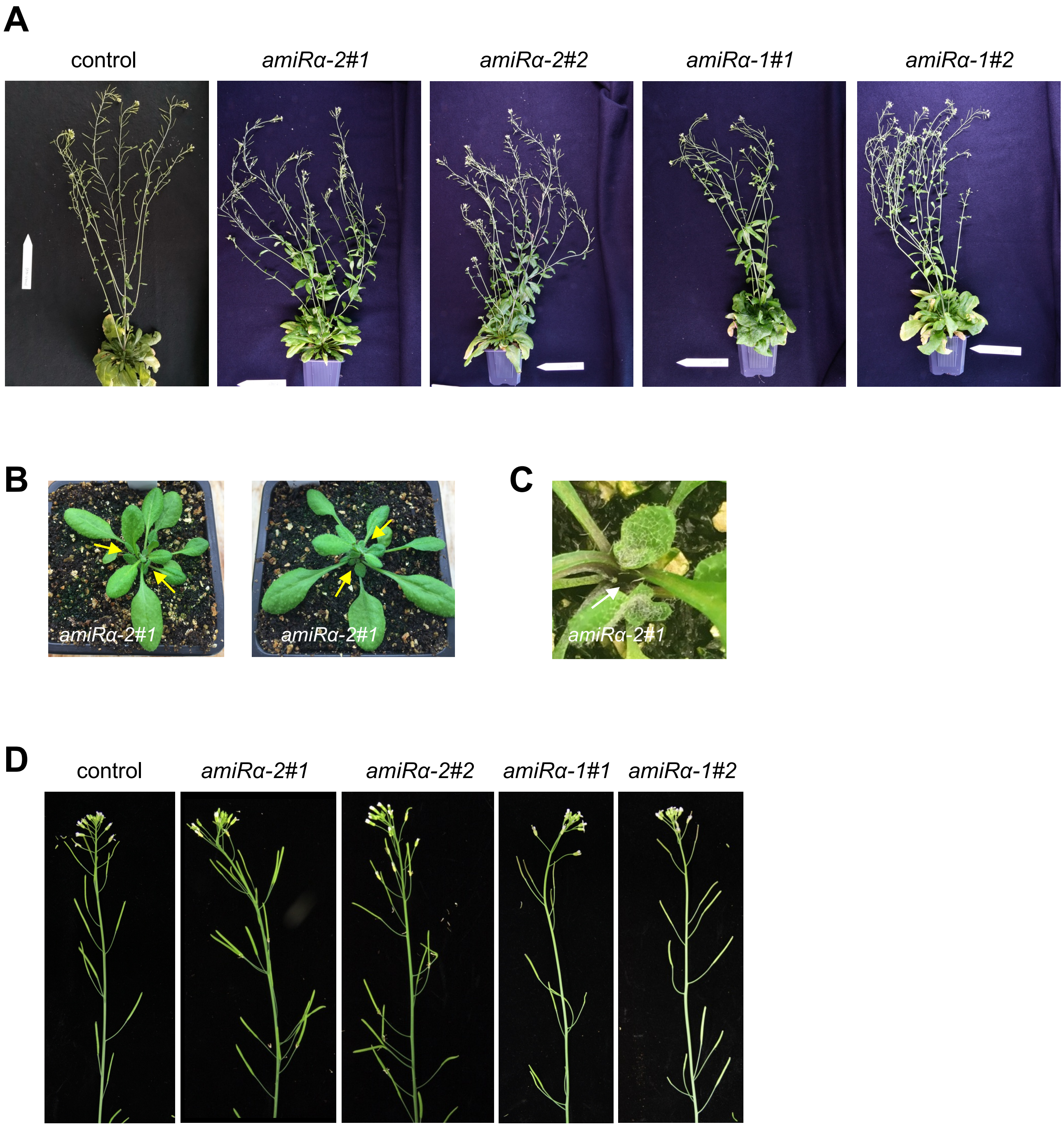

Supplementary Figure S6. Silencing of *SnRK1α* in the SAM compromises meristem function and plant architecture. A, Representative images of plants expressing *STM-VENUS* (control) together with *pSTM::amiRα-1* or *pSTM::amiRα-2* and grown under equinoctial conditions until the completion of flowering. **B-C**, Representative images of 22d-old plants of the most severely affected *amiRα* line (*amiRα-2*#1) showing activation of axillary meristems before flowering (yellow arrows in **B-C**) and termination of the main meristem (white arrow in **C**). **D,** Representative images of plants expressing *STM-VENUS* together with *pSTM::TFP-N7* (control), *pSTM::amiRα-1,* or *pSTM::amiRα-2,* and grown under short-day conditions for 3 weeks and then transferred to long-day conditions until the completion of flowering. #1 and #2 denote two independent lines for the indicated *amiRs.*

Supplementary Figure S7

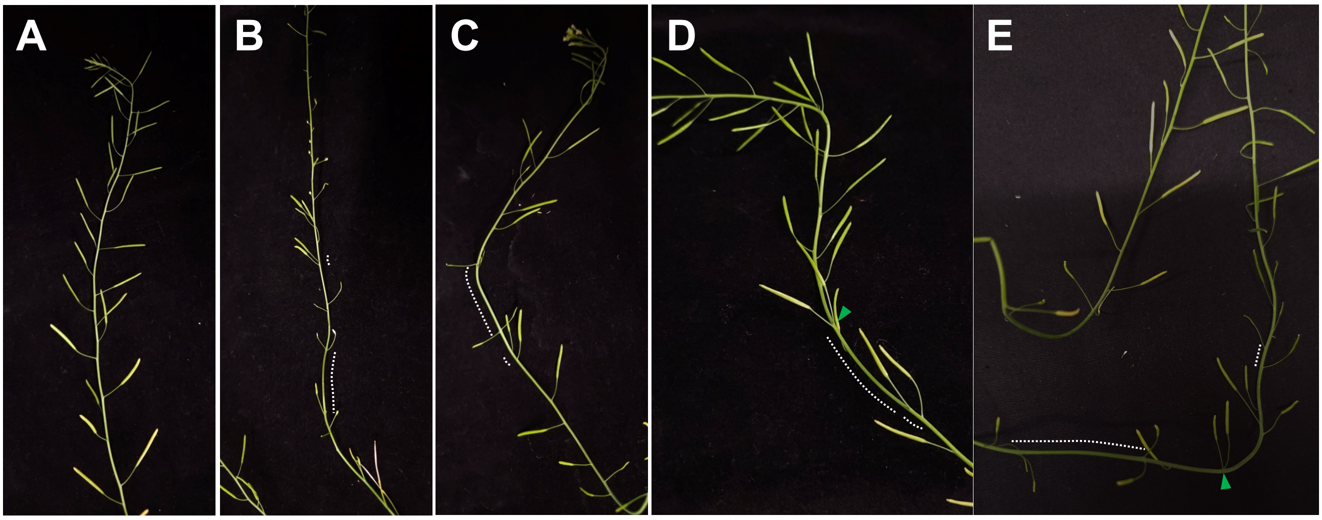

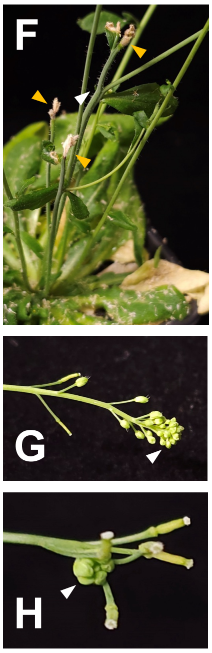

I

Line	Irregular Internodes	Pedicel Fusion	Total Plants
Control	28,57%	0%	7
pRPS5a:amiRNA-1#1	50%	0%	2
pRPS5a:amiRNA-1#2	100%	83,3%	6
pRPS5a:amiRNA-1#3	100%	85,7%	7
pRPS5a:amiRNA-2#1	71,4%	14,3%	7
pRPS5a:amiRNA-2#2	100%	16,6%	6
pRPS5a:amiRNA-2#3	100%	57,14%	7
pFD:amiRNA-1#1	100%	100%	5
pFD:amiRNA-1#2	100%	100%	8
pFD:amiRNA-1#3	100%	87,5%	8
pFD:amiRNA-2#1	100%	0%	5
pFD:amiRNA-2#2	87,5%	25%	8
pFD:amiRNA-2#3	100%	71,4%	7

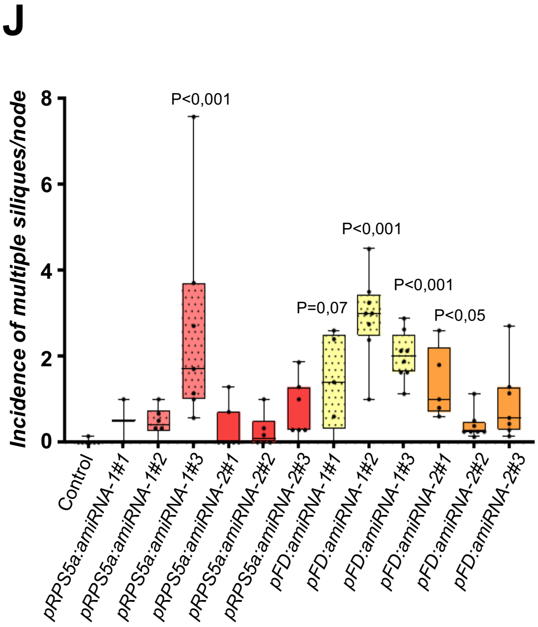

Supplementary Figure S7. Silencing *SnRK1α* in the SAM using alternative promoters also compromises meristem function and plant architecture. A-E, Representative image of control plants (**A**) and four independent lines expressing *amiRα-1* and *amiRα-2* under two different promoters: *pRPS5::amiRα-1* (**B**), *pRPS5::amiRα-2* (**C**), *pFD::amiRα-1* (**D**) and *pFD::amiRα-2* (**E**). Phenotyping was performed with T2 plants. **F-G**, Representative images of the most severely affected *amiRα* line (*pFD::amiRα-1*) showing senescence of the inflorescence meristems (**F,** orange arrow) and gross growth defects (fasciation) in the stem (**F**, white arrow) and flower organs (**G-H**, white arrows). **I**, Frequency of plants showing aberrant internode elongation (dotted white lines in **B-E**) and pedicel fusion (green arrows in **D-E**) in the main inflorescence stem. **J**, Frequency of nodes with two or more siliques (total number of main inflorescence nodes harboring two or more siliques in the indicated genotype divided by the total number of plants scored). *p*-values are shown for statistically significant differences between the indicated lines to the control (Kruskal-Wallis with Dunn´s test).

SUPPLEMENTARY INFORMATION

Plant material

For generating the *pSTM::STM-VENUS/pSTM::TFP-N7* line, the 5.7 kb promoter of *STM* was amplified by PCR and introduced in a pDONR-P4-P1R plasmid by Gateway recombination cloning (1). A triple LR reaction was then performed between this vector, a TFP-N7 recombined with a pDONR221, a Nos-T7 terminator recombined with a pDONR-P2R-P3, and a pH7m34GW destination vector. The resulting gene construct was then introduced into *Arabidopsis* plants already expressing the *STM-VENUS* reporter using *Agrobacterium*. Transformants were selected based on the hygromycin resistance of the pH7m34GW vector. For generating the *pSTM::amiRα* lines, two amiRNAs targeting both *SnRK1α1* and *SnRK1α2* (*amiRα-1* and *amiRα-2*) were designed using the WMD3 Web microRNA designer tool (2) and introduced into the primary *miRNA319a* (At4g23713) backbone in the pHBT95 vector (3). The resulting primary *miRNA319a*, harboring the *amiRα-1* or *amiRα-2* sequence, was used to generate the corresponding *amiRα-1* and *amiRα-2* pENTRY 1_2 constructs and recombined with *pSTM* (5.7 kb) pENTRY 4_1r and *NOS-T7*-pENTRY 2r_3 into pK7m34GW (4). The resulting construct was transformed into the *STM-VENUS* line and single-insertion homozygous progeny was selected on kanamycin. For generating the *pRPS5::amiRα* and *pFD::amiRα* lines, the *pRPS5a* (1.7 kb) and *pFD* (3 kb) promoters were cloned into a pDONR_P4_P1r vector and the resulting pENTRY 4_1r clones were recombined with *amiRα-1* and *amiRα-2* pENTRY 1_2 and *NOS-T7*-pENTRY 2r_3 constructs into pK7m34GW. The resulting construct was transformed into the *SnRK1α1-GFP* line and single-insertion homozygous progeny was selected on kanamycin.

Plant growth conditions

Seeds were sown in excess into individual pots (7 x 7 x 6 cm, W/L/H) with soil (Levington F2) that were thereafter kept in a 4°C room for three days before being transferred to growth cabinets. The pots were covered with transparent plastic lids during the first five days and the plants were thinned out seven days after sowing to leave one plant per pot. For most experiments, seedlings were initially grown in short-day conditions (8 h/16 h light/dark period; 22°C/18°C) for 3-4 weeks and then transferred to continuous light (24 h light, temperature: 22°C) and kept under an irradiance of 60 (LL) or 170 (HL) µmol m^{-2} s^{-1}, provided by white fluorescent lamps. Unless

otherwise indicated, plants were grown in HL conditions. For dark treatment, plants grown under HL were put into the dark for 24, 48 or 72 h. For the assays with excised inflorescences and STM-VENUS imaging of *amiRα* lines, seedlings were grown in short-day conditions (8 h/16 h light/dark period; 22°C/18°C) for 3-4 weeks and then transferred to long-day conditions (16 h/8 h light/dark period; 22°C/18°C).

For experiments involving SAM imaging, gene expression or protein analyses, plants were grown in the indicated conditions until bolting, after which SAMs were dissected at the beginning of the flowering stage when the main inflorescence reached 3-5 cm in height.

For phenotyping the *amiRα* lines, seeds were germinated, and plants grown under equinoctial conditions (12 h/12 h light/dark period; 100-110 µmol m^{-2} s^{-1}; 22°C/18°C). Phenotypes were scored when flowering was completed [stage 6.90; (5)].

SAM imaging and quantification

Meristems were imaged in water using a 20X long-distance water-dipping objective mounted either on a LSM880 (Zeiss; www.zeiss.com) or a SP8 (Leica; www.zeiss.com) confocal microscope. Z-stacks of 1-2 µm spacing were taken and the spacing was kept constant within a single experiment.

Confocal Z stacks were analyzed using the ImageJ software (https://fiji.sc/) and a custom-made code written in Matlab (Mathworks Inc., Natick, MA). ImageJ was used for generating the panel figures showing the fluorescence reporters in the SAMs. To generate the panels, z-projections (sum slices) of meristems expressing TFP, GFP or VENUS were performed, and the Fire color code was used to represent the signal. The expression levels of the different fluorescence reporters were analyzed by using the Matlab code (see https://gitlab.com/slcu/teamHJ/pau/RegionsAnalysis). *pTCSn::GFP* reporter was analyzed by using a pipeline (6), which measures total fluorescence intensity of circular expression domains. STM-VENUS signal and TFP signal were analyzed as follows . Firstly, a z-sum intensity projection was performed followed by a gaussian blur with a smoothing kernel with standard deviation sigma = 5 µm. Second, a region of interest (SAM core excluding the emerging floral organs) was drawn on the image projection and the mean intensity of the region was extracted. The mean intensity was chosen as a measure of the STM-VENUS and TFP levels instead of the

total intensity to minimize the influence of manually drawing the region of interest and the new buds on the signal.

Protein extraction, quantification, and immunoblotting

For extraction of total protein for the immunoblot analyses, one leaf or complete inflorescence meristems (five per replicate) were ground in liquid N_2. Finely ground tissue (approximately 30-40 mg per sample) was extracted in 1.5 volumes of buffer [150 mM NaCl, 1% (v/v) Triton-X, 50 mM Tris-HCl (pH 8.0), 3 mM DTT, supplemented with 50 µM MG132, 1:20 Complete EDTA-free Protease Inhibitor Cocktail (Roche), and 1:500 Phosphatase Inhibitor Cocktails 2 and 3 (Sigma-Aldrich; www.sigmaaldrich.com)]. Homogenates were cleared by centrifugation at 21130 x g for 15 minutes at 4°C, and supernatants were used for total protein quantification (Pierce 660 nm protein assay reagent; www.thermofisher.com). Single use aliquots of 40 µg total protein were prepared and denatured in Laemmli buffer(7) before storage at -20°C. Total protein samples were separated by SDS-PAGE in 10% acrylamide gels, transferred to PVDF membranes (wet transfer at 4°C, 100 V, 70 min) and probed using 1:1000 dilutions of STM, p-AMPK, SnRK1α1 or TUBULIN antibodies (see Table S1 for further details).

Chemiluminescent detection was performed using a 1:10,000 dilution of Peroxidase AffiniPure goat anti-rabbit IgG (H+L) (Jackson ImmunoResearch; www.jacksoniummuno.com), and SuperSignal West Femto Maximum Sensitivity Substrate (Thermo Scientific).

Band intensity was quantified using ImageJ. The bands of interest were selected with the rectangle tool and the corresponding intensity peaks were plotted. The peaks were separated using the line tool after which their area was measured. For quantifying STM-VENUS and SnRK1α1 amounts, band intensity values were normalized to the values corresponding to their respective Rubisco band in the Ponceau-stained membrane. For quantifying SnRK1α phosphorylation, band intensity values of the phospho-SnRK1α immunoblot were normalized to the values corresponding to their respective SnRK1α1 immunoblot.

RNA extraction, cDNA synthesis and qRT-PCR

Total RNA was extracted from approximately 30-40 mg of finely ground tissue, with the Plant Nucleospin RNA extraction kit (Macherey-Nagel; www.mn-net.com), according to the

manufacturer's instructions. DNAse-treated RNA (1 µg) was used for synthesis of cDNA libraries, using SuperScript III Reverse Transcriptase (Life Technologies;www.thermofisher.com).

qRT-PCR analyses were performed in 384-well reaction plates using the QuantStudio™ 7 Flex Real- Time PCR System (Thermo Fisher). The reactions were prepared in a total volume of 10 µL containing 1 µL of cDNA (diluted 1:10) corresponding to 2.5 ng of RNA, 5 µL of iTaq Universal SYBR Green Supermix (BioRad www.bio-rad.com) and 0.8 µL of each gene-specific 5 µM primer. No-template and -RT controls were included for each gene in comparative gene expression analyses.

The $2^{-\Delta\Delta Ct}$ method was used for relative quantification. Expression values were normalised to the geometric mean of Ct values obtained for the following reference genes: *UBQ10* (At4g05320) and *UBC21* (At5g25760). Expression of reference genes, *STM* (At1g62360), *HB25* (At5g65410), *AIL7* (At5g65510), *DIN10* (At5g20250), *DRM2* (At2g33830) and *SEN5* (At3g15450) was assessed using the primers described in Table S1.

Sugar measurements

Soluble sugars were extracted from aliquots (15-20 mg) of frozen tissue powder using chloroform-methanol as described in (8). Sucrose, glucose and fructose were quantified by high-performance hydrophilic interaction chromatography on a 150 x 2.1 mm Luna Omega Sugar (Phenomenex Inc.; Aschaffenburg, Germany; www.phenomenex.com) column, fitted with a 4 x 2.0 mm Security Guard Cartridge (Phenomenex) and coupled to a QTrap 5500 triple quadrupole mass spectrometer (AB Sciex, Foster City, USA; sciex.com). Samples were spiked with ^{13}C-labelled internal standards for correction of ion suppression and other matrix effects (9). The column was maintained at 25°C and equilibrated with 90 % (v/v) acetonitrile, 5 % (v/v) isopropanol, 5 % (v/v) water (eluent A) before injection of samples (1 µl). Sugars were eluted (flow rate 0.25 ml min-1) with the following gradient, obtained by mixing eluent A with eluent B [5 % (v/v) acetonitrile, 25 % (v/v) isopropanol, 70 % (v/v) water): 0 – 2.5 min [10 % B]; 2.5 - 25 min [10 - 30 % B]; 25 – 30.5 min [30 % B]; 30.5 – 31 min [30 – 10 % B]; 31 – 40 min [10 % B]. Sugars were quantified by tandem mass spectrometry using the parent/product ion transitions as described in (9). Tre6P was quantified in chloroform-methanol extracts as described in (8) with modifications as described in (10).

Y2H assays

For Y2H assays, a pGBKT7 construct harboring the *SnRK1α1* coding sequence had previously been generated (11). The *STM* coding sequence was amplified from seedling cDNA and cloned into pDONR221 and the resulting pENTRY-STM construct was recombined into a Gateway-compatible pDEST-GADT7 vector (12). Y2H assays were performed as described (13). To test for interaction between SnRK1α1 and STM, yeast cells were co-transformed with the pGBKT7-SnRK1α1 construct, harboring the GAL4 DNA-binding domain (BD), and the pDEST-GADT7-STM construct, harboring the GAL4 activation domain (AD). As controls, pGBKT7-SnRK1α1 subunit and pDEST-GADT7-STM constructs were co-transformed with the respective complementary empty vectors. Yeast growth was assessed in low-stringency media [-Leucine (L)/-Tryptophan(W)] for selection of co-transformants, and in higher-stringency media [-L/-W/-Histidine (H)/] for selection of interactors.

Protoplast assays

Protoplast transient expression assays were carried out as previously described, using freshly isolated mesophyll cells from mature fully expanded Arabidopsis leaves (14, 15). For the expression of SnRK1α1 (WT or SnRK1α1^{K48M}) with a C-terminal HA tag, previously described pHBT95-based constructs were used (14). For expressing STM with a C-terminal HA tag, the *STM* CDS was amplified from *Arabidopsis* seedling cDNA, introducing BamHI (5') and StuI (3') restriction sites for cloning into the pHBT vector (15). For expressing STM with a C-terminal GFP tag, a pENTRY-STM construct was recombined with the p2GWC7 vector (4). For generating a reporter of STM activity (*pCUC1::LUC*), the *CUC1* promoter (1623 bp upstream of the ATG start codon) was amplified from genomic DNA and cloned into pDONR221. The resulting pENTRY-pCUC1 construct was recombined into the pGWL7 vector harboring the *LUC* gene (4). STM activity was monitored using *pCUC1::LUC* and a *pUBQ10::GUS* reporter as transfection control. A plasmid yielding no protein expression was used as a negative control and to adjust the total amount of DNA per transfection to 20 μg. Effector and reporter constructs (SnRK1α1, STM, and *pCUC1::LUC*) were added at a 1:1:2 ratio, respectively. For all samples, the LUC signal was normalized by the GUS signal to account for potential differences in transfection efficiency (15). For co-IP assays, pHBT95-SnRK1α1-HA and p2GWC7-STM-GFP were used in a 1:1 ratio to co-transfect 1 ml of mesophyll cell protoplasts. Following transfection, protoplasts were incubated

for 16h under light conditions (25-30 μmol m^{-2} s^{-1} and 23°C), and then harvested by centrifugation and flash frozen.

Frozen cell pellets were lysed in 500 μl of lysis buffer [150 mM NaCl, 0.5% (v/v) Triton-X, 50 mM Tris-HCl (pH 8.0), 3 mM DTT, supplemented with 50 μM MG132, 1:20 Complete EDTA-free Protease Inhibitor Cocktail (Roche), and 1:500 Phosphatase Inhibitor Cocktails 2 and 3 (Sigma-Aldrich)]. The cleared lysate was incubated with 30 μl of super-paramagnetic μMAC beads coupled to a monoclonal anti-GFP antibody (Miltenyi Biotec) for 2h at 4°C with gentle rocking. Purified immunocomplexes were eluted in Laemmli buffer, boiled, run in an 10% SDS-PAGE gel, transferred to PVDF membranes (wet transfer at 4°C, 100 V, 70 min) and analyzed by immunoblotting using antibodies against GFP (1/1000, Roche, #11814460001), HA (1/1000, Roche, #11867423001), and SnRK1α1 (16), used at a 1:2000 dilution.

For immunodetection, secondary antibodies conjugated with horseradish peroxidase (AffiniPure goat anti-rabbit (#111035144) or anti-rat IgG (H+L) (#112035167); Jackson ImmunoResearch; www.jacksoniummuno.com) were used at 1:10000 dilutions. Chemiluminescence-based detection of peroxidase activity was performed using a SuperSignal West Femto Maximum Sensitivity Substrate (Thermo Scientific).

References

1. B. Landrein, *et al.*, Mechanical stress contributes to the expression of the STM homeobox gene in Arabidopsis shoot meristems. *Elife* **4** (2015).
2. R. Schwab, S. Ossowski, M. Riester, N. Warthmann, D. Weigel, Highly Specific Gene Silencing by Artificial MicroRNAs in *Arabidopsis*. *Plant Cell* **18**, 1121–1133 (2006).
3. C. Martinho, *et al.*, Dissection of miRNA pathways using Arabidopsis mesophyll protoplasts. *Mol Plant* **8**, 261–275 (2015).
4. M. Karimi, A. Depicker, P. Hilson, Recombinational Cloning with Plant Gateway Vectors. *Plant Physiol* **145**, 1144–1154 (2007).
5. D. C. Boyes, Growth Stage-Based Phenotypic Analysis of Arabidopsis: A Model for High Throughput Functional Genomics in Plants. *Plant Cell* **13**, 1499–1510 (2001).
6. P. Formosa-Jordan, B. Landrein, "Quantifying Gene Expression Domains in Plant Shoot Apical Meristems" in *Methods in Molecular Biology*, (2023), pp. 537–551.
7. U. K. Laemmli, Cleavage of structural proteins during the assembly of the head of bacteriophage T4. *Nature* **227**, 680–685 (1970).
8. J. E. Lunn, *et al.*, Sugar-induced increases in trehalose 6-phosphate are correlated with redox activation of ADPglucose pyrophosphorylase and higher rates of starch synthesis in Arabidopsis thaliana. *Biochemical Journal* **397**, 139–148 (2006).
9. R. Feil, J. E. Lunn, "Quantification of Soluble Sugars and Sugar Alcohols by LC-MS/MS" in *Methods in Molecular Biology*, (2018), pp. 87–100.

10. C. M. Figueroa, *et al.*, Trehalose 6-phosphate coordinates organic and amino acid metabolism with carbon availability. *The Plant Journal* **85**, 410–423 (2016).
11. A. Rodrigues, *et al.*, ABI1 and PP2CA phosphatases are negative regulators of Snf1-related protein kinase1 signaling in Arabidopsis. *Plant Cell* **25**, 3871–3884 (2013).
12. P. Rossignol, S. Collier, M. Bush, P. Shaw, J. H. Doonan, *Arabidopsis* POT1A interacts with TERT-V(I8), an N-terminal splicing variant of telomerase. *J Cell Sci* **120**, 3678–3687 (2007).
13. A. Saez, A. Rodrigues, J. Santiago, S. Rubio, P. L. Rodriguez, HAB1-SWI3B interaction reveals a link between abscisic acid signaling and putative SWI/SNF chromatin-remodeling complexes in Arabidopsis. *Plant Cell* **20** (2008).
14. E. Baena-González, F. Rolland, J. M. Thevelein, J. Sheen, A central integrator of transcription networks in plant stress and energy signalling. *Nature* **448**, 938–942 (2007).
15. A. Confraria, E. Baena-González, *Using Arabidopsis protoplasts to study cellular responses to environmental stress* (2016).
16. B. Belda-Palazón, *et al.*, A dual function of SnRK2 kinases in the regulation of SnRK1 and plant growth. *Nat Plants* **6**, 1345–1353 (2020).

Table S1. List of all primers used in this study

qRT-PCR	Name	Sequence	
STM (At1g62360)	STM_F	TCGACTTCTTCCTCGGATGACCCA	
	STM_R	TCTCCGGTTATGGAGAGACAGCAA	
HB25 (At5g65410)	HB25_F	ACGGCTGAGCAAAAGGAAAG	
	HB25_R	GATGAAGTGGTGAAGGCGAC	
AIL7 (At5g65510)	AIL7_F	CGGAACCTTTGCAACCGAA	
	AIL7_R	AGAAGAGGACGGAAGCAACT	
DIN10 (At5g20250)	DIN10_F	GATTCTTCGTGCTCGACTCC	
	DIN10_R	TTAGCAAGCTGACACCATCAC	
SEN5 (At3g15450)	SEN5_F	GCGAAACTCTCTCCGACTTC	
	SEN5_R	CCACAGAACAACCTTTGACG	
DRM2 (At2g33830)	DRM2_F	CTTCGACAAGCCTTCTCACC	
	DRM2_R	TCGTCGCTGTATAGCCAATC	
UBQ10 (At4g05320)	UBQ10_F	GGCCTTGTATAATCCCTGATGAATAAG	
	UBQ10_R	AAAGAGATAACAGGAACGGAAACATAGT	
UBC21 (At5g25760)	UBC21_F	CTGCGACTCAGGGAATCTTCTAA	
	UBC21_R	TTGTGCCATTGAATTGAACCC	
**Cloning**	**Name**	**Sequence**	**Description**
amiRNAα-1	amiRNAα-1_1	gaTGATAGCAACCTTATGCGCTGtctctcttttgtattcc	Generation of amiRNAα-1 and cloning into pHBT
	amiRNAα-1_2	gaCAGCGCATAAGGTTGCTATCAtcaaagagaatcaatga	
	amiRNAα-1_3	gaCAACGCATAAGGTAGCTATCTtcacaggtcgtgatatg	
	amiRNAα-1_4	gaAGATAGCTACCTTATGCGTTGtctacatatatattcct	
amiRNAα-2	amiRNAα-2_1	gaTACTGAAGTCCAAGAGCGCATtctctcttttgtattcc	Generation of amiRNAα-2 and cloning into pHBT
	amiRNAα-2_2	gaATGCGCTCTTGGACTTCAGTAtcaaagagaatcaatga	
	amiRNAα-2_3	gaATACGCTCTTGGAGTTCAGTTtcacaggtcgtgatatg	
	amiRNAα-2_4	gaAACTGAACTCCAAGAGCGTATtctacatatatattcct	
Gateway - amiRNAα-1/amiRNAα-2	amiRNAα-attB1	GGGGACAAGTTTGTACAAAAAAGCAGGCTTACAAACACACGCTCGGACG	GW cloning of amiR1011 from pHBT to pDONR221
	amiRNAα-attB2	GGGGACCACTTTGTACAAGAAAGCTGGGTCCATGGCGATGCCTTAAATAAAG	
Gateway - STM	STM -attB1	GGGGACAAGTTTGTACAAAAAAGCAGGCTTAATGGAGAGTGGTTCCAACAG	GW cloning of STM to pDONR221
	STM -attB2	GGGGACCACTTTGTACAAGAAAGCTGGGTTAAGCATGGTGGAGGAGATGTG	
STM	STM_BamHI	CGGGATCCATGGAGAGTGGTTCC	Cloning of STM into a pHBT vector with a C-terminal HA tag
	STM_StuI	AAGGCCTAAGCATGGTGGAGGAG	
Gateway - pCUC1	pCUC1 _5_UTR_attB1	GGGGACAAGTTTGTACAAAAAAGCAGGCTTAcaagtctcttaaattgtg	GW cloning of CUC1 promoter to pGWL7
	pCUC1 _5_UTR_attB2	GGGGACCACTTTGTACAAGAAAGCTGGGTTAtgtcggcacaagaagaaagc	
promoter FD	pFD_GW_Fw	GGGGACAACTTTGTATAGAAAAGTTGttctacacctaaaagtctaagacg	GW cloning of FD promoter (3 kb) to pDONR_P4_P1r
	pFD_GW_Rv	GGGGACTGCTTTTTTGTACAAACTTGtggaaaagagaacagaagtgaacc	
promoter RPS5a	pRPS5_GW_Fw	GGGGACAACTTTGTATAGAAAAGTTGcacaatgagagaggaactgtagg	GW cloning of RPS5a promoter (1.7 kb) to pDONR_P4_P1r
	pRPS5_GW_Rv	GGGGACTGCTTTTTTGTACAAACTTGggctgtggtgagagaaacag	

Table S2. List of all previously published lines used in this study		
**PLANT LINE**	**REFERENCE**	
35S::SnRK1α1 (*SnRK1α1-OE*)	Jossier et al., 2009 Plant J	
pSTM::STM-VENUS	Heisler et al., 2005 Curr Biol	The STM promoter corresponds to 5.7 kb
pTCSn::GFP	Zürcher et al., 2013 Plant Phys	
proSnRK1α1::SnRK1α1-GFP/snrk1α1-3 ("SnRK1α1-GFP")	Crozet et al., 2015 Plant J	

Table S3. List of all antibodies used in this study							
**ANTIBODY**	**Antibody - full description**	**Source**	**Company**	**Product number**		**Dilution**	**Dilution buffer**
Anti-SnRK1α1	Anti-SnRK1α1	Custom-made	Belda-Palazón et al., 2020	Belda-Palazón et al., 2020	Rabbit polyclonal	1:4000	1% w/v nonfat dry milk in 1X TBS
Anti-phospho-AMPKα (T172)	Phospho-AMPKalpha (Thr172) (40H9) Antibody Ab#1	Commercial	Cell Signaling	2535	Rabbit monoclonal	1:1000	1% w/v nonfat dry milk in 1X TBS
Anti-STM	Anti-STM (Homeobox protein SHOOT MERISTEMLESS)	Commercial	Agrisera	AS11 1764	Rabbit polyclonal	1:1000	1% w/v nonfat dry milk in 1X TBS
Anti-TUBULIN	Arabidopsis β-Tubulin (F-1)	Commercial	Santa Cruz Biotechnology, Inc.	sc-166729	Mouse monoclonal	1:1000	1% w/v nonfat dry milk in 1X TBS
Anti-mouse-PRX	Peroxidase Affinipure goat anti-mouse IgG (H+L), Secondary antibody	Commercial	Jackson ImmunoResearch Lab, inc	115035146	Goat polyclonal	1:20000	1% w/v nonfat dry milk in 1X TBS
Anti-rabbit-PRX	Peroxidase Affinipure goat anti-rabbit IgG (H+L), Secondary antibody	Commercial	Jackson ImmunoResearch Lab, inc	111035144	Goat polyclonal	1:20000	1% w/v nonfat dry milk in 1X TBS
